# Simple Equations Method (SEsM): Algorithm, Connection with Hirota Method, Inverse Scattering Transform Method, and Several Other Methods

**DOI:** 10.3390/e23010010

**Published:** 2020-12-23

**Authors:** Nikolay K. Vitanov, Zlatinka I. Dimitrova, Kaloyan N. Vitanov

**Affiliations:** 1 Institute of Mechanics, Bulgarian Academy of Sciences, Acad. G. Bonchev Str., Block 4, 1113 Sofia, Bulgaria; kalovitanov@gmail.com; 2 Institute of Solid State Physics, Bulgarian Academy of Sciences, Blvd. Tzarigradsko Chaussee 72, 1784 Sofia, Bulgaria; zdim@issp.bas.bg

**Keywords:** nonlinear partial differential equations, exact solutions, Simple Equations Method (SEsM), Hirota method, inverse scattering transform method, homogeneous balance method, extended homogeneous balance method, auxiliary equation method, Jacobi elliptic function expansion method, F-expansion method, modified simple equation method, trial function method, first integral method

## Abstract

The goal of this article is to discuss the Simple Equations Method (SEsM) for obtaining exact solutions of nonlinear partial differential equations and to show that several well-known methods for obtaining exact solutions of such equations are connected to SEsM. In more detail, we show that the Hirota method is connected to a particular case of SEsM for a specific form of the function from Step 2 of SEsM and for simple equations of the kinds of differential equations for exponential functions. We illustrate this particular case of SEsM by obtaining the three- soliton solution of the Korteweg-de Vries equation, two-soliton solution of the nonlinear Schrödinger equation, and the soliton solution of the Ishimori equation for the spin dynamics of ferromagnetic materials. Then we show that a particular case of SEsM can be used in order to reproduce the methodology of the inverse scattering transform method for the case of the Burgers equation and Korteweg-de Vries equation. This particular case is connected to use of a specific case of Step 2 of SEsM. This step is connected to: (i) representation of the solution of the solved nonlinear partial differential equation as expansion as power series containing powers of a “small” parameter ϵ; (ii) solving the differential equations arising from this representation by means of Fourier series, and (iii) transition from the obtained solution for small values of ϵ to solution for arbitrary finite values of ϵ. Finally, we show that the much-used homogeneous balance method, extended homogeneous balance method, auxiliary equation method, Jacobi elliptic function expansion method, F-expansion method, modified simple equation method, trial function method and first integral method are connected to particular cases of SEsM.

## 1. Introduction

Nature and human society are rich sources of complex systems (examples can be found, e.g., in economics, social sciences, network theory, dynamics of research groups, etc. [1,2,3,4,5,6,7]). Because of this the complex systems attract much research attention in the last decades, see for examples, [8,9,10,11,12,13,14,15,16,17,18,19,20,21,22]. Most of the complex systems are nonlinear—many such examples can be found in the fluid mechanics or solid-state physics [23,24,25,26,27,28,29]. The effects connected to the nonlinearity can be studied, for example, by means of time series analysis or by means of models based on differential or difference equations (additional information about the methodology of the nonlinear time series analysis, some applications of this methodology and basic information about nonlinear differential equations, can be seen, e.g., in [30,31,32,33,34,35,36,37,38,39,40,41,42,43,44,45]). Very often the used model equations are nonlinear partial differential equations, and because of this the methodology for solving such equations is interesting to the researchers. Many years ago the methodology for obtaining exact solutions of nonlinear partial differential equations was connected to transformations that transform the solved nonlinear partial differential equation to a linear differential equation. One example is the Hopf-Cole transformation [46,47] which transforms the nonlinear Burgers equation to the linear heat equation. An appropriate transformation of the Korteweg-de Vries equation connected this equation to the famous equation of Schrödinger and led to the development of the Method of inverse scattering transform [48,49,50]. Almost at the same time, Hirota developed a method for obtaining exact solutions of NPDEs—Hirota method [51,52]. This method is based on the bilinearization of the solved nonlinear partial differential equation after appropriate transformation of the nonlinearity of the equation. Truncated Painleve expansions may lead to many of these appropriate transformations [53,54,55,56,57]. We note the work of Kudryashov who formulated the Method of Simplest Equation (MSE) [58] based on determination of singularity order *n* of the solved NPDE and on searching of a particular solution of this equation as series containing powers of solutions of a simpler equation called the simplest equation. The methodology was extended [59] and applied for obtaining traveling wave solutions of nonlinear partial differential equations (see, e.g., [60,61,62]). We write several more words on the work of Kudryashov as it is of interest for our discussion below in the text and the Method of Simplest Equation leads to interesting results. Kudryashov [63] used various transformations in order to transform the nonlinearity of a generalized evolution equation of the wave dynamics and to obtain exact solutions of this equation. This research was continued in [57,64,65] and recent results connected to the application of the Method of Simplest Equation can be found, for example, in [66,67,68,69,70].

Recently we presented an algorithm for obtaining exact and approximate solutions of nonlinear partial differential equations called Simple Equations Method (SEsM) [71,72,73,74]. We shall discuss this algorithm and its connection to other methods below in this text. We note that some elements of the methodology can be seen in our articles written almost 30 years ago [75,76,77,78,79,80]. More than 10 years ago [81,82] we have used the ordinary differential equation of Bernoulli as simplest equation [83] and applied methodology called Modified Method of Simplest Equation to ecology and population dynamics [84]. In these publications we have used the concept of the balance equation. The Modified Method of Simplest Equation—MMSE [85,86]—is based on the determination of the kind of the simplest equation and truncation of the series of solutions of the simplest equation by means of application of a balance equation and it is equivalent of the Method of Simplest Equation mentioned above. Up to 2018 our contributions to the methodology and its application have been connected to the MMSE [87,88,89,90,91,92,93,94,95]. We note especially the article [94] where we have extended the methodology of the MMSE to simplest equations of the class

(1) $${\left(\frac{d^{k}g}{d\xi^{k}}\right)}^{l}=\sum_{j=0}^{m}d_{j}g^{j}$$

where k=1,…, l=1,…, and *m* and $d_{j}$ are parameters. Equation (1) contains as particular cases, for example: (i) trigonometric functions; (ii) hyperbolic functions; (iii) elliptic functions of Jacobi; (iv) elliptic function of Weierstrass.

In the course of time, we extended the algorithm of the Modified Method of Simplest Equation. Here we are going to discuss the last version which is connected to the possibility of use of more than one simple equation. This modification is called SEsM—Simple Equations Method. The reason for the use of this name is that the used simple equations are more simple than the solved nonlinear partial differential equation, but these simple equations in fact can be quite complicated. Thus we have to substitute the word “simplest” by the word “simple”. A variant of SEsM based on two simple equations was applied in [96] and the first description of the algorithm was made in [71] and then in [72,73,74]. For more applications of particular cases of the algorithm see [97,98].

We note that SEsM is not a universal algorithm for obtaining exact analytical solutions of nonlinear differential equations. With very large probability there are many exact solutions of nonlinear differential equation which can not be obtained by SEsM. The presence of such solutions is of large interest for us as it shows possible ways for further development of SEsM. We note also that some steps of SEsM are not rigidly fixed. Examples are the choice of transformations in Step 1 of SEsM and the selection of the simple equation in Step 5. This flexibility increases the number of equations which can be treated by SEsM but the set of these equations is still a subclass of the class of nonlinear differential equations that possess exact solutions.

The organization of the text below is as follows. We describe SEsM in Section 2. In Section 3 we show that the famous method of Hirota is connected to a particular case of SEsM and then we use the connection between SEsM and Hirota method in order to show that SEsM can lead to solutions of integrable differential equations: examples are the three-solution solution of the Korteweg-de Vries equation, two-soliton solution of the nonlinear Schrödinger equation and the soliton solution of the Ishimori equation. In Section 4 we discuss the connection between SEsM and the inverse scattering transform method. This connection is shown in the examples of the Burgers equation and the Korteweg-de Vries equation. In Section 5 we formulate several assumptions which show that numerous methods for obtaining exact particular solutions of nonlinear partial differential equations, namely, homogeneous balance method, extended homogeneous balance method, auxiliary equation method, Jacobi elliptic function expansion method, F-expansion method, modified simple equation method, trial function method, first integral method, are connected to particular cases of SEsM. Generalizations of some of these methods are formulated. Several concluding remarks are summarized in Section 6.

## 2. Simple Equations Method (SEsM)

**Figure 1:**
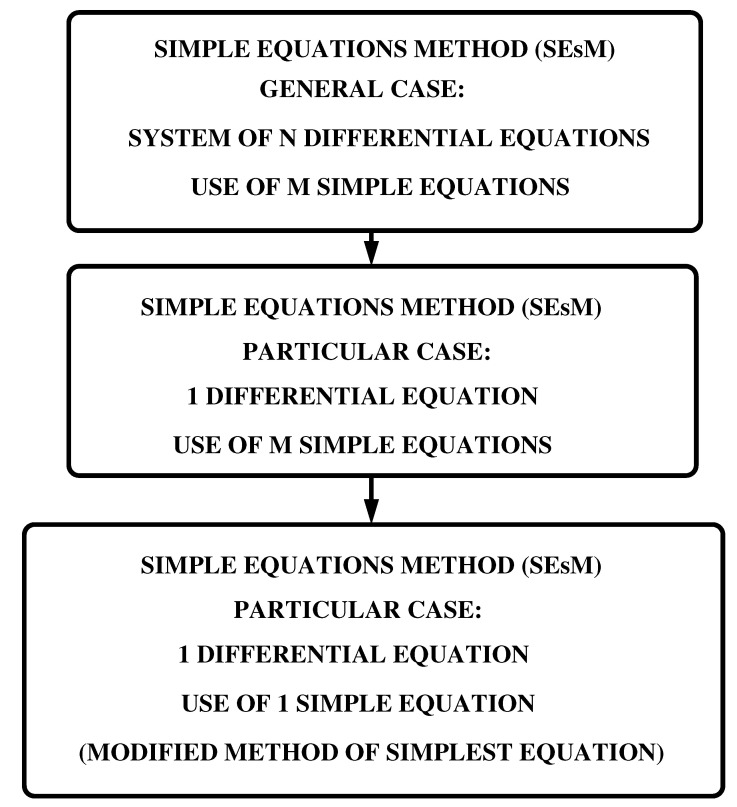
The general case of simple equations method (SEsM) and its particular cases. The general case of the SEsM is for a system of *N* differential equations and the solution is constructed on the basis of solutions of *M* simple equations (we note that he parameter *M* may depend on the parameter *N*). A particular case of the general SEsM is the case when one has to solve one differential equation and the solution is constructed on the basis of solutions of *M* simple equations. The simplest case of SEsM is when one has to solve one differential equation and the solution is constructed by solutions of one simple equation. This particular case is known as the Modified Method of Simplest Equation.

Simple Equations Method (SEsM) is an algorithm for obtaining exact and approximate solutions of nonlinear differential equations. In general (Figure 1) the algorithm is designed to obtain solutions of systems of *N* nonlinear differential equations by the use of solutions of *M* simple equations. The most applications of the algorithm up to now are for obtaining solutions of 1 nonlinear differential equations by the use of solutions of (a) one simple equation or (b) more than one simple equation (see, e.g., [83,84,85,86,87,88,89,90,91,92,93,94,95] where the corresponding particular case of SEsM called Modified Method of Simplest Equation is applied for obtaining exact solutions of numerous nonlinear partial differential equations). The development of SEsM started with the use of a solution of one simple equation in order to obtain a solution of one nonlinear differential equation. This version of the algorithm was called Modified Method of Simplest Equation. We shall show below that SEsM is capable to lead to particular exact solutions of nonlinear differential equations. In addition, we shall show that SEsM is connected to the method of Hirota and to the inverse scattering transform method and because of this SEsM can lead not only to particular solutions of some nonlinear differential equations.

**Figure 2:**
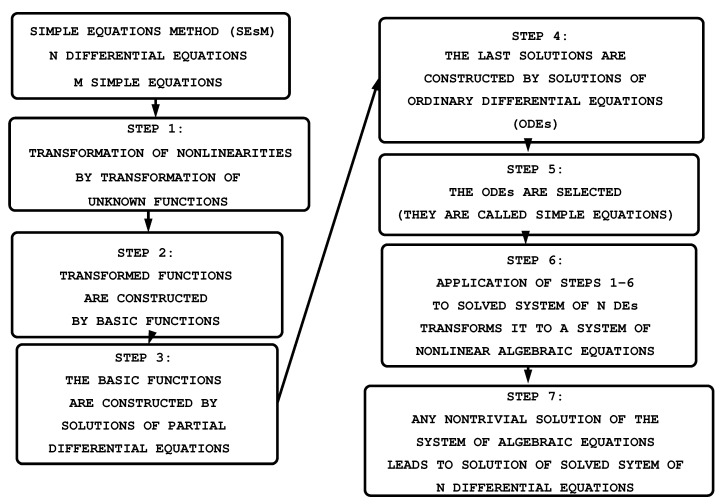
The seven steps in the general case of SEsM. For a more detailed description, see the text.

SEsM has seven steps which are shown in Figure 2.

We consider a system of nonlinear partial differential equations

(2) $$A_{i}\left[u_{1}\left(x,\ldots ,t\right),\ldots ,u_{n}\left(x,\ldots ,t\right)\right]=0,\;\;\;i=1,\ldots ,n,$$

where $A_{i}\left[u_{1}\left(x,\ldots ,t\right),\ldots ,u_{n}\left(x,\ldots ,t\right),\ldots \right]$ depend on the functions $u_{1}\left(x,\ldots ,t\right),\ldots ,$$u_{n}\left(x,\ldots ,t\right)$ and some of their derivatives ($u_{i}$ can be a function of more than 1 spatial coordinates). Then we proceed as follows.

**(1.)**  We apply transformations
  (3) $$u_{i}\left(x,...,t\right)=T_{i}\left[F_{i}\left(x,\ldots ,t\right),G_{i}\left(x,\ldots ,t\right),\ldots \right],$$
  where $T_{i}\left(F_{i},G_{i},\ldots \right)$ is some function of other functions $F_{i},G_{i},\ldots$. In general $F_{i}\left(x,\ldots ,t\right)$, $G_{i}\left(x,\ldots ,t\right)$, … are functions of several spatial variables as well as of the time. The transformation has the goal to transform the nonlinearity of the solved differential equations to more treatable kind of nonlinearity or the transformation may even remove the nonlinearity. In the case of one solved equation the transformation T(F,G,…) can be: the Painleve expansion; $u\left(x,t\right)=4\tan^{-1}\left[F\left(x,t\right)\right]$ in the case of the sine–Gordon equation; $u\left(x,t\right)=4\tanh^{-1}\left[F\left(x,t\right)\right]$ in the case of sh-Gordon (Poisson–Boltzmann equation) (for applications of the last two transformations, see, e.g., [75,76,77,78]); $u\left(x,t\right)=\frac{F(x,t)}{G(x,t)}$; $u\left(x,t\right)=\frac{\sum_{i=0}^{I}a_{i}{\left[F\left(x,t\right)\right]}^{i}}{\sum_{j=0}^{J}b_{j}{\left[G\left(x,t\right)\right]}^{j}}$; or another transformation.
  In numerous particular cases, one may skip this step (then we have just $u_{i}\left(x,...,t\right)=F_{i}\left(x,...,t\right)$) but in many cases the step is necessary for obtaining a solution of the studied nonlinear PDE. The application of (3) to (2) leads to a nonlinear PDEs for the functions $F_{i},G_{i},\ldots$.
  We note that no general form of the transformations $T_{i}$ is known up to now and because of this we cannot write a general relationships for these transformations here. Moreover, some equations can be treated without such transformations. The transformations allow us to extend the class of equations for which exact solutions can be obtained by means of SEsM.
**(2.)**  The functions $F_{i}\left(x,...,t\right)$, $G_{i}\left(x,\ldots ,t\right)$, … are represented as a function of other functions $f_{i1},...,f_{iN}$, $g_{i1},\ldots ,g_{iM}$, …. The functions *f* and *g* are connected to solutions of some differential equations (these equations can be partial or ordinary differential equations) which are more simple than Equation (2). We note that the possible values of *N* and *M* are *N* = 1, 2, …, *M* = 1, 2, … (there may be an infinite number of functions f too). The forms of the functions $F_{i}\left(f_{1},\ldots ,f_{N}\right)$, $G_{i}\left(x,\ldots ,t\right)$, … can be different. For an example for the case of a single solved equation the function *F* can have the form
  (4) $$\begin{array}{c} F=\alpha +\sum_{i_{1}=1}^{N}\beta_{i_{1}}f_{i_{1}}+\sum_{i_{1}=1}^{N}\sum_{i_{2}=1}^{N}\gamma_{i_{1},i_{2}}f_{i_{1}}f_{i_{2}}+\sum_{i_{1}=1}^{N}\ldots \sum_{i_{N}=1}^{N}\sigma_{i_{1},\ldots ,i_{N}}f_{i_{1}}\ldots f_{i_{N}}, \end{array}$$
  where $\alpha ,\beta_{i_{1}},\gamma_{i_{1},i_{2}},\sigma_{i_{1},\ldots ,i_{N}}\ldots$ are parameters. Of course $F(f_{1},\ldots ,f_{N})$ can have another form too (i.e., the form of *F* can be different from (4)). SEsM is very flexible with respect to the form of $F_{i}$, $G_{i}$, …. We note that the relationship (4) contains, as a particular case, the relationship used by Hirota [51]. The power series $\sum_{i=0}^{N}\mu_{n}f^{n}$ (where μ is a parameter) used in the previous versions of the methodology based on one simple equation (i.e., the Modified Method of Simplest Equation) are also a particular case of the relationship (4).
**(3.)**  In general the functions used in $F_{i},G_{i},\ldots$ - the functions $f_{i1},\ldots ,f_{iN}$, $g_{i1},\ldots ,g_{iM}$ are solutions of some partial differential equations. These equations are more simple than the solved nonlinear partial differential equation. There are two possibilities: (i) one may use solutions of the simple partial differential equations if such solutions are available, or (ii) one transforms the more simple partial differential equations by means of appropriate ansätze (e.g., traveling-wave ansätze such as $\xi =\hat{\alpha }x+\hat{\beta }t$; $\zeta =\hat{\mu }y+\hat{\nu }t;\ldots$). Then the solved differential equations for $f_{i1}$, …, $f_{iN}$, $g_{i1},\ldots ,g_{iM}$, … may be reduced to differential equations $E_{l}$, containing derivatives of one or several functions
  (5) $$E_{l}\left[a\left(\xi \right),a_{\xi },a_{\xi \xi },\ldots ,b\left(\zeta \right),b_{\zeta },b_{\zeta \zeta },\ldots \right]=0;\;\;l=1,\ldots ,N+M+\ldots .$$
  In many cases (e.g., if the equations for the functions $f_{1},\ldots$ are ordinary differential equations) one may skip this step, but the step may be necessary if the equations for $f_{1},\ldots$ are complicated partial differential equations.
**(4.)**  We assume that the functions a(ξ), b(ζ), etc., are functions of other functions, such as, v(ξ), w(ζ), etc., e.g,
  (6) a(ξ)=A[v(ξ)];b(ζ)=B[w(ζ)];….
  Note that SEsM does not prescribe the forms of the functions *A*, *B*, …. Thus, different relationships are possible. Often one uses a finite-series relationship, for example,
  (7) $$a\left(\xi \right)=\sum_{\mu_{1}=-\nu_{1}}^{\nu_{2}}q_{\mu_{1}}{\left[v\left(\xi \right)\right]}^{\mu_{1}};\;\;\;b\left(\zeta \right)=\sum_{\mu_{2}=-\nu_{3}}^{\nu_{4}}r_{\mu_{2}}{\left[w\left(\zeta \right)\right]}^{\mu_{2}},\ldots .$$
  where $q_{\mu_{1}}$, $r_{\mu_{2}}$, … are parameters. However, other kinds of relationships, and more complicated ones, are also possible.
**(5.)**  The functions v(ξ), w(ζ), … are solutions of simple ordinary differential equations. For about 10 years we have used the particular case of the described methodology that was based on the use of just one simple equation. This simple equation was called the simplest equation and the methodology based on one equation was called the Modified Method of Simplest Equation. SEsM contains the Modified Method of Simplest Equation as a particular case.
**(6.)**  The application of the steps 1–5 to Equation (2) transforms the left-hand side of these equations. The results of this transformation can be functions which are sums of terms where each term contains some function multiplied by a coefficient. This coefficient contains some of the parameters of the solved equations and some of the parameters of the solutions. In most cases, a balance procedure must be applied in order to ensure that the above-mentioned relationships for the coefficients contain more than one term (e.g., if the result of the transformation is a polynomial, then the balance procedure has to ensure that the coefficient of each term of the polynomial is a relationship that contains at least two terms). This balance procedure may lead to one or more additional relationships among the parameters of the solved equation and parameters of the solution. These relationships are known as balance equations.
**(7.)**  We may obtain a nontrivial solution of Equation (2) if all coefficients mentioned in Step 6 are set to 0. This condition usually leads to a system of nonlinear algebraic equations for the coefficients of the solved nonlinear PDE and for the coefficients of the solution. Any nontrivial solution of this algebraic system leads to a solution the studied nonlinear partial differential equation. Usually, the above system of algebraic equations contains many equations and because of this, the support of a computer algebra system is needed.

**Figure 3:**
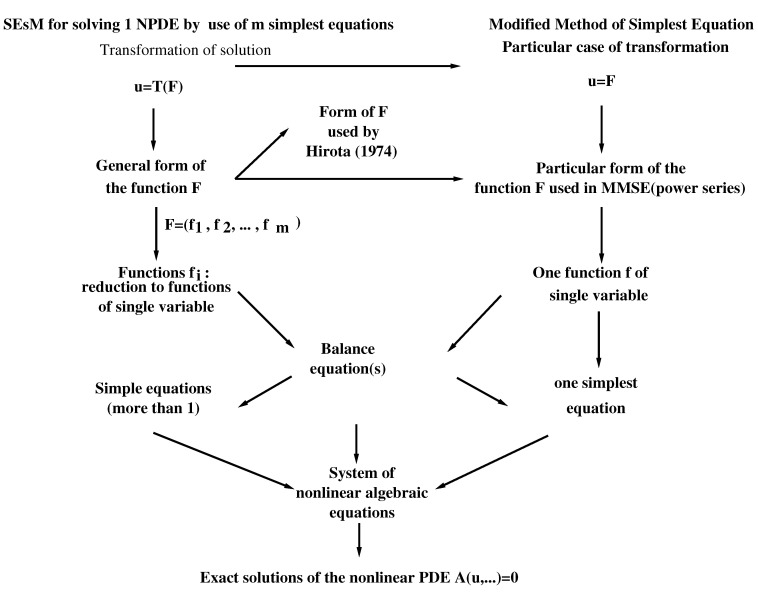
The steps for the particular case of SEsM for solving one differential equation by means of *m* simple equations. On the right-hand side of the figure one sees the particular case of the methodology called the Modified Method of Simplest Equation.

Figure 3 shows the most frequently used particular cases of SEsM: the cases when one searches for a solution of one nonlinear differential equation (the cases are: (i) solutions of m>1 simple equations are used, or (ii) the solution of just one simple equation is used). In these cases, we search for solution *u* of the solved equation and at step 1 of SEsM we transform *u* by means of a transformation *T*. The solved equation is transformed into an equation for the function *F* and at the following steps of SEsM *F* is represented by functions $f_{i}$ which are constructed by the known solutions of the used simple equations. Thus the solved nonlinear differential equation is reduced to a system of nonlinear algebraic equations and each nontrivial solution of this system leads to a particular solution of the solved differential equation. In the most simple case of SEsM (the Modified Method of Simplest Equation) we skip the Step 1 (the one with the transformation *T*) and *F* is represented as a power series of the solution *f* of a single simple equation. The substitution of these power series in the solved equation reduces this equation to a system of nonlinear algebraic equations and each nontrivial solution of this system leads to an exact particular solution of the solved nonlinear differential equation.

Below we age going to discuss the relation among SEsM and several much-used methods for obtaining exact solutions of nonlinear partial differential equations.

## 3. Hirota Method and SEsM

### 3.1. Hirota Method

Hirota method is a very popular method for obtaining soliton solutions of integrable nonlinear partial differential equations. One of the first applications of the method was in the famous article of Hirota [51] and detailed description of the method and its applications can be found in the book of Hirota [52]. Here, we present a very brief summary of this method which is as follows. At Step 1 of the method, one makes a transformation of the solved nonlinear partial differential equation. Hirota often used the transformation

(8) $$u\left(x,t\right)=2\frac{\partial^{2}}{\partial x^{2}}f\left(x,t\right)=2\left(\frac{f\frac{\partial^{2}f}{\partial x^{2}}-{\left(\frac{\partial f}{\partial x}\right)}^{2}}{f^{2}}\right).$$

The function *f* is called sometimes the τ-function. At Step 2 of the method, one searches for a solution of the obtained after the transformation equation in the form

(9) $$f=\alpha +\epsilon f_{1}+\epsilon^{2}f_{2}+\epsilon^{3}f_{3}+\ldots$$

where α and ϵ are parameters. At Step 3 of the method, (9) is substituted in the solved nonlinear partial differential equation. At Step 4 of the method, the obtained equations for the orders ϵ, $\epsilon^{2}$, $\epsilon^{3}$, … are solved. In Step 5 of the method, the exact solution is constructed on the basis of the solutions for ϵ, $\epsilon^{2}$, $\epsilon^{3}$, ….

In order to deal with Step 4 of his method, Hirota introduced the famous bilinear operators,

(10) $$\begin{array}{c} \begin{array}{ccc} D_{t}^{n}\left(a.b\right) & = & {\left(\frac{\partial }{\partial t}-\frac{\partial }{\partial t^{\prime }}\right)}^{n}a\left(x,t\right)b\left(x^{\prime },t^{\prime }\right)\;\;\mathrm{at}\;t^{\prime }=t,\\ D_{x}^{m}\left(a.b\right) & = & {\left(\frac{\partial }{\partial x}-\frac{\partial }{\partial x^{\prime }}\right)}^{m}a\left(x,t\right)b\left(x^{\prime },t^{\prime }\right)\;\;\mathrm{at}\;x^{\prime }=x,\\ D_{x}^{m}D_{t}^{n}\left(a.b\right) & = & {\left(\frac{\partial }{\partial x}-\frac{\partial }{\partial x^{\prime }}\right)}^{m}{\left(\frac{\partial }{\partial t}-\frac{\partial }{\partial t^{\prime }}\right)}^{n}a\left(x,t\right)b\left(x^{\prime },t^{\prime }\right)\;\;\mathrm{at}\;t^{\prime }=t;\;x^{\prime }=x, \end{array} \end{array}$$

which can be written also as follows [99]

(11) $$\begin{array}{ccc} D_{x}^{m}\left(a.b\right) & = & \sum_{j=0}^{m}\frac{{\left(-1\right)}^{\left(m-j\right)}m!}{j!(m-j)!}\frac{\partial^{j}a}{\partial x^{j}}\frac{\partial^{m-j}b}{\partial x^{m-j}},\\ D_{x}^{m}D_{t}^{n}\left(a.b\right) & = & \sum_{j=0}^{m}\sum_{i=0}^{n}\frac{{\left(-1\right)}^{\left(m+n-j-i\right)}m!}{j!(m-j)!}\frac{n!}{i!(n-i)!}\frac{\partial^{i+j}a}{\partial t^{i}\partial x^{j}}\frac{\partial^{m+n-i-j}b}{\partial t^{n-i}\partial x^{m-j}}. \end{array}$$

The bilinear operators have useful properties such as

(12) $$D_{x}^{m}\left(a.1\right)=\frac{\partial^{m}a}{\partial x^{m}}$$

(13) $$D_{x}^{m}\left(a.b\right)={\left(-1\right)}^{m}D_{x}^{m}\left(b.a\right)$$

(14) $$D_{x}^{m}\left(a.a\right)=0\;\;\;\mathrm{for}\;\;m\;\;\mathrm{odd}$$

(15) $$\begin{array}{c} D_{x}^{m}D_{t}^{n}\left(\exp\left[k_{1}x-\omega_{1}t\right].\exp\left[k_{2}x-\omega_{2}t\right]\right)={\left(k_{1}-k_{2}\right)}^{m}{\left(\omega_{2}-\omega_{1}\right)}^{n}\exp\left[\left(k_{1}+k_{2}\right)x-\left(\omega_{1}+\omega_{2}\right)t\right]. \end{array}$$

By means of the bilinear operators in many cases, one can find a solution of the sequence of equations for the orders ϵ, $\epsilon^{2}$, $\epsilon^{3}$, … without much effort.

### 3.2. Hirota Method and SEsM

**Assumption** **1.** *The method of Hirota is connected to a particular case of SEsM when the transformation in step 1 of SEsM is the same as the transformation used in Hirota’s method, the representation of function f by means of $f_{1},f_{2},\ldots$ from Step 2 of SEsM is (9), the differential equations for $f_{1},f_{2},\ldots$ from Step 3 of SEsM are the chain of equations obtained for the orders ϵ, $\epsilon^{2}$, … within the scope of Hirota’s method and the simple equations which are used in the construction of the solutions for $f_{1},f_{2},\ldots$ are differential equations for exponential functions.*

We consider SEsM and impose restrictions on its steps in order to reduce this method to a particular case connected to the Hirota method. We proceed as follows. First, we consider the particular case of SEsM where, in Step 1, the transformation (8) is used or any other transformation used within the scope of Hirota’s method. We note that these transformations are a small part of the possible transformations which can be used in SEsM. At Step 2 of SEsM we consider again a particular case: we use the relationship (9) in order to represent the function *f* by means of the functions $f_{1},f_{2},\ldots$. This is a particular case from the point of view of SEsM as many other kinds of representations of *f* by $f_{1},f_{2},\ldots$ are possible in SEsM. At Step 3 of SEsM, we use one more particular case by considering differential equations for $f_{1},f_{2},\ldots$ to be exactly the equations that are obtained within the scope of application of Hirota’s method. This is a particular case as many other kinds of differential equations can be used in SEsM. At Step 4 of SEsM we consider the relationships between $f_{i}$ and the more simple functions to be exactly these ones which arise when Hirota’s method is used. From the point of view of SEsM this is a particular case as much more kinds of relationships can be used in SEsM and one example for this is a relationship of the kind (4). At Step 5 of SEsM we have to determine the simple equations for the functions which participate in the construction of function $f_{i}$. We take the particular case when these functions are solutions of very simple equations namely differential equations for exponential functions. We note that much more complicated functions can be used in SEsM at this step such as Jacobi elliptic functions, for example. At Step 6 of SEsM we substitute the relationships for $f_{i}$ in the corresponding differential equations and perform a balance procedure if needed. This will lead to a system of nonlinear algebraic equations. We note that in the case of Korteweg-de Vries equation such balance procedure is not needed. Finally, at Step 7 of SEsM one solves the system of nonlinear algebraic equations and obtains the solution of the corresponding nonlinear PDE.

Thus, by means of particular cases of procedures of SEsM we have reduced SEsM to a particular case connected to the Hirota’s method.

Now on the basis of the above, we shall show that SEsM can lead to soliton and multisoliton solutions of several famous equations.

### 3.3. Example 1: The Three-Soliton Solution of the Korteweg-de Vries Equation

We consider the Korteweg-de Vries equation

(16) $$\frac{\partial u}{\partial t}+6u\frac{\partial u}{\partial x}+\frac{\partial^{3}u}{\partial x^{3}}=0.$$

At Step 1 of SEsM we transform the nonlinearity in (16). This is made by the transformation (8). The result is

(17) $$f\frac{\partial^{2}f}{\partial x\partial t}+\frac{\partial f}{\partial x}\frac{\partial f}{\partial t}+f\frac{\partial^{4}f}{\partial x^{4}}-4\frac{\partial f}{\partial x}\frac{\partial^{3}f}{\partial x^{3}}+3{\left(\frac{\partial^{2}f}{\partial x^{2}}\right)}^{2}=0,$$

and this can be written by means of the bilinear operators of Hirota as

(18) $$\left(D_{x}D_{t}+D_{x}^{4}\right)\left(f\cdot f\right)=0$$

At Step 2 of SEsM we represent the function *f* by means of functions $f_{1},f_{2},\ldots$ which will be constructed by means of solutions of the simple equations. This representation is (9). At Step 3, of SEsM we substitute (9) in (17) and obtain the sequence of differential equations for $f_{1}$, $f_{2}$, … as follows

(19) $$2\frac{\partial }{\partial x}\left(\frac{\partial }{\partial t}+\frac{\partial^{3}}{\partial x^{3}}\right)f_{1}=0.$$

Note that this is a linear equation. The equations for $f_{2}$ and $f_{3}$ are

(20) $$2\frac{\partial }{\partial x}\left(\frac{\partial }{\partial t}+\frac{\partial^{3}}{\partial x^{3}}\right)f_{2}=-D_{x}\left(D_{t}+D_{x}^{3}\right)\left(f_{1}\cdot f_{1}\right),$$

(21) $$2\frac{\partial }{\partial x}\left(\frac{\partial }{\partial t}+\frac{\partial^{3}}{\partial x^{3}}\right)f_{3}=-D_{x}\left(D_{t}+D_{x}^{3}\right)\left(f_{1}\cdot f_{2}+f_{2}\cdot f_{1}\right).$$

The equations for $f_{4}$, $f_{5}$, … are obtained in a similar way. At steps 4 and 5 of SEsM we have to represent the functions $f_{i}$, i=1,2,… by means of other functions which are solutions of our simple equations. The form of the corresponding relationships depends on the particular solution of (19) we start with. In order to obtain the single-soliton solution of the Korteweg-de Vries equation, we are starting with a solution for $f_{1}$ constructed by just one function which is solution of a very simple differential equation: the differential equation for exponential function

(22) $$\frac{dg_{1}}{d\eta_{1}}=g_{1};\;\;g_{1}=\exp\left(\eta_{1}\right),$$

where $\eta_{1}=\lambda_{1}x+\omega_{1}t+\sigma_{1}$ and $\lambda_{1}$, $\omega_{1}$ and $\sigma_{1}$ are parameters. We choose $f_{1}$ just as

(23) $$f_{1}=\exp\left(\eta_{1}\right)$$

The substitution of (23) in (19) leads to the algebraic equation (the dispersion relation)

(24) $$\omega_{1}+\lambda_{1}^{3}=0.$$

We note that no balance procedure is required here (i.e., Step 6 of SEsM can be skipped). The substitution of $f_{1}$ in (20) leads to 0 in the right-hand side of this equation. Then $f_{2}$ can be taken to be 0. The same is the situation with $f_{3},f_{4},\ldots$. Thus we obtain the following solution for *f*

(25) $$f=1+\epsilon f_{1}.$$

The parameter ϵ can be absorbed in $\sigma_{1}$ and the solution for *f* leads to the one-soliton solution for *u* by use of (8).

In order to obtain the two-soliton solution of the Korteweg-de Vries equation, we take the solution of (19) constructed by solutions of two simple equations

(26) $$\frac{dg_{1}}{d\eta_{1}}=g_{1};\;\;g_{1}=\exp\left(\eta_{1}\right);\;\;\;\;\frac{dg_{2}}{d\eta_{2}}=g_{2};\;\;g_{2}=\exp\left(\eta_{2}\right),$$

where $\eta_{i}=\lambda_{i}x+\omega_{i}t+\sigma_{i}$ and $\lambda_{i}$, $\omega_{i}$ and $\sigma_{i}$, i=1,2 are parameters. Now we construct $f_{1}$ as the sum of the solutions of the two simple equations

(27) $$f_{1}=\exp\left(\eta_{1}\right)+\exp\left(\eta_{2}\right).$$

The substitution of (27) in (19) leads to a system of two algebraic equations, namely

(28) $$\omega_{i}+\lambda_{i}^{3}=0,\;\;i=1,2$$

The substitution of (27) in (20) leads to a nonlinear equation for $f_{2}$. The solution of this equation is

(29) $$f_{2}=a_{12}\exp\left(\eta_{1}+\eta_{2}\right),$$

where $a_{12}=\frac{{\left(\lambda_{1}-\lambda_{2}\right)}^{2}}{{\left(\lambda_{1}+\lambda_{2}\right)}^{2}}$. The substitution of $f_{2}$ in (21) leads to 0 in the right-hand side of this equation. Thus we can choose $f_{3}=0$ and then $f_{4}=f_{5}=\cdots =0$. The solution for *f* becomes

(30) $$f=1+\epsilon \left[\exp\left(\eta_{1}\right)+\exp\left(\eta_{2}\right)\right]+\epsilon^{2}a_{12}\exp\left(\eta_{1}+\eta_{2}\right).$$

Again the ϵ and $\epsilon^{2}$ can be absorbed by $\sigma_{1,2}$ and $a_{1,2}$ and the application of (8) leads to the two-soliton solution of the Korteweg-de Vries equation.

In order to obtain the three-soliton solution of the Korteweg-de Vries equation, we start with the solution for $f_{1}$ constructed by means of solutions of three simple equations for exponential functions,

(31) $$\frac{dg_{i}}{d\eta_{i}}=g_{i};\;\;g_{i}=\exp\left(\eta_{i}\right);\;\;i=1,2,3,$$

where $\eta_{i}=\lambda_{i}x+\omega_{i}t+\sigma_{i}$ and $\lambda_{i}$, $\omega_{i}$ and $\sigma_{i}$, i=1,2,3 are parameters.Then we present $f_{1}$ as the sum of the solutions of the three simple equations

(32) $$f_{1}=\exp\left(\eta_{1}\right)+\exp\left(\eta_{2}\right)+\exp\left(\eta_{3}\right).$$

The substitution of (32) in (19) leads to the algebraic relationships (dispersion relations)

(33) $$\omega_{i}+\lambda_{i}^{3}=0,\;\;i=1,2,3,$$

and the substitution of (32) in (20) leads to the following relationship for $f_{2}$

(34) $$f_{2}=a_{12}\exp\left(\eta_{1}+\eta_{2}\right)+a_{13}\exp\left(\eta_{1}+\eta_{3}\right)+a_{23}\exp\left(\eta_{2}+\eta_{3}\right),$$

where

$$a_{ij}=\frac{{\left(\lambda_{i}-\lambda_{j}\right)}^{2}}{{\left(\lambda_{i}+\lambda_{j}\right)}^{2}},\;\;i,j=1,2,3,\;\;i<j.$$

The substitution of the obtained solutions for $f_{1}$ and $f_{2}$ in (21) leads to the following solution for $f_{3}$

(35) $$f_{3}=b_{123}\exp\left(\eta_{1}+\eta_{2}+\eta_{3}\right),\;\;b_{123}=a_{12}a_{13}a_{23}$$

$f_{3}$ is a single exponential function again and then the right-hand side of the equation for $f_{4}$ is 0 and we can take $f_{4}=0$. Then we can continue with $f_{5}=f_{6}=\cdots =0$ and the obtained solution for *f* is

(36) $$\begin{array}{c} f=1+\epsilon \left[\exp\left(\eta_{1}\right)+\exp\left(\eta_{2}\right)+\exp\left(\eta_{3}\right)\right]+\epsilon^{2}[a_{12}\exp\left(\eta_{1}+\eta_{2}\right)+\\ a_{13}\exp\left(\eta_{1}+\eta_{3}\right)+a_{23}\exp\left(\eta_{2}+\eta_{3}\right]+\epsilon^{3}b_{123}\exp\left(\eta_{1}+\eta_{2}+\eta_{3}\right) \end{array}$$

and after the absorption of epsilons and application of (8) we obtain the three-soliton solution of the Korteweg-de Vries equation.

The procedure can be easily continued and the *N*-soliton solution of the Korteweg-de Vries equation can be obtained by means of a particular case of the SEsM methodology. We use *N* simple equations for exponential functions and begin by solution $f_{1}$ which is a sum of the solutions of these simple equations. Then step by step we obtain $f_{i}$ up to i=N and for i>N we can set $f_{i}=0$ and then we can construct the *N*-soliton solution of the Korteweg-de Vries equation.

### 3.4. Example 2: The Two-Soliton Solution of the Nonlinear Schrödinger Equation

Now we show how SEsM leads to the two-soliton solution of the nonlinear Schrödinger equation. Here the transformation at Step 1 of SEsM leads to equations for two functions *F* and *G*. The connection of SEsM with the Hirota’s method makes this task of obtaining the two-soliton solution quite easy. The nonlinear Schrödinger equation is

(37) $$i\frac{\partial \psi }{\partial t}+\frac{\partial^{2}\psi }{\partial x^{2}}+q|\psi {|}^{2}\psi =0,$$

where *q* is a parameter. We consider the case q<0 (and we shall set below q=−2 for convenience) with boundary conditions $|\psi {|}^{2}=\rho_{0}^{2}$ at x→±∞. At Step 1 of SEsM we transform the nonlinearity in (37) by the transformation

(38) $$\psi =\frac{G}{F}.$$

Note that the transformation (38) is different in comparison to the transformation in the case of the Korteweg-de Vries equation. This is an illustration of the feature of SEsM to allow for different kinds of transformations at Step 1 of application of the methodology.

The substitution of (38) to (37) leads to (* means complex conjugated quantity and $D_{x}$ and $D_{t}$ are the operators of Hirota, described above in the text)

(39) $$i\frac{D_{t}G\cdot F}{F^{2}}+\frac{D_{x}^{2}G\cdot F}{F^{2}}-\frac{G}{F}\frac{D_{x}^{2}F\cdot F}{F^{2}}-2\frac{G}{F}\frac{GG^{*}}{F^{2}}=0.$$

This can be written as

(40) $$\left\{i\left[D_{t}+D_{x}^{2}\right]G\cdot F\right\}F^{2}-GF\left[D_{x}^{2}F\cdot F-2GG^{*}\right]=0.$$

Introducing the constant λ we can write two coupled equations of the basis of (39)

(41) $$[iD_{t}+D_{x}^{2}]G\cdot F=\lambda GF$$

(42) $$D_{x}^{2}+2GG^{*}=\lambda F^{2}$$

λ is a constant which will be determined below. At Step 2 of SEsM we represent *F* and *G* by functions which will be then constructed by solutions of simple equations (differential equations for exponential functions in the case discussed here). These representations are made by the following expansions

(43) $$F=1+\epsilon f_{1}+\epsilon^{2}f_{2}+\ldots ,$$

(44) $$G=g_{0}\left(1+\epsilon g_{1}+\epsilon^{2}g_{2}+\ldots \right)$$

$f_{1},f_{2},\ldots ,g_{1},g_{2},\ldots$ have to go to 0 at x→−∞. Then at x→−∞$g_{o}g_{0}^{*}=\rho_{0}^{2}$ and from (43) and (44) at x→−∞

(45) $$\left[iD_{t}+D_{x}^{2}-\lambda \right]g_{0}\cdot 1=0,$$

(46) $$\left[D_{x}^{2}-\lambda \right]1\cdot 1=-2g_{0}g_{0}^{*},$$

one obtains

(47) $$\lambda =2\rho_{0}^{2};\;g_{0}=\rho_{0}\exp\left(i\eta \right);\;\;\theta =kx-\omega t;\;\;\omega =k^{2}+2\rho_{0}^{2}.$$

Above, *k* is a real constant. The substitution of (43) and (44) in (41) and (42) leads to relationships for the different powers of ϵ as follows. For the terms of order of ϵ

(48) $$\left[i\left(D_{t}+2kD_{x}\right)+D_{x}^{2}\right]\left(g_{1}\cdot 1+1\cdot f_{1}\right)=0$$

(49) $$\left[D_{x}^{2}-2\rho_{0}^{2}\right]\left(f_{1}\cdot 1+1\cdot f_{1}\right)=-2\rho_{0}^{2}\left(g_{1}+g_{1}^{*}\right).$$

For the terms of order of $\epsilon^{2}$

(50) $$\left[i\left(D_{t}+2kD_{x}\right)+D_{x}^{2}\right]\left(g_{2}\cdot 1+g_{1}\cdot f_{1}+1\cdot f_{2}\right)=0$$

(51) $$\left[D_{x}^{2}-2\rho_{0}^{2}\right]\left(f_{2}\cdot 1+f_{1}\cdot f_{1}+1\cdot f_{2}\right)=-2\rho_{0}^{2}\left(g_{2}+g_{1}+g_{1}^{*}+g_{2}^{*}\right).$$

For the terms of order of $\epsilon^{3}$

(52) $$\left[i\left(D_{t}+2kD_{x}\right)+D_{x}^{2}\right]\left(g_{3}\cdot 1+g_{2}\cdot f_{1}+g_{1}\cdot f_{2}+1\cdot f_{3}\right)=0$$

(53) $$\left[D_{x}^{2}-2\rho_{0}^{2}\right]\left(f_{3}\cdot 1+f_{2}\cdot f_{1}+f_{1}\cdot f_{2}+1\cdot f_{3}\right)=-2\rho_{0}^{2}\left(g_{3}+g_{2}+g_{1}^{*}+g_{1}g_{2}^{*}+g_{3}^{*}\right).$$

Next we write relationships if the functions $f_{1},\ldots ,g_{1},\ldots$ by means of functions which are solutions of simple equations. (48) and (49) have many possible solutions. The most simple ones are constructed by means of a simple function which is a solution of a very simple differential equation: the differential equation for exponential function (Steps 3–5 of SEsM). These solutions are

(54) $$f_{1}=\exp\left(\eta \right);\;\;g_{1}=b\exp\left(\eta \right),$$

where η=qx−ωt and for the satisfaction of (48) and (49) the following relationships must hold (there is no need of balance procedure—Step 6. of SEsM and the relationships follows from the system of algebraic equations obtained from the substitution of (53) in (48) and (49)—Step 7 of SEsM)

(55) $$\omega =q\left(2k-\sqrt{4\rho_{0}^{2}-q^{2}}\right);\;\;b=-\frac{q^{2}+i\left(\omega -2kq\right)}{q^{2}-i\left(\omega -2kq\right)}.$$

This choice of $f_{1}$ and $g_{1}$ leads to $f_{2}=f_{3}=\ldots 0$ and $g_{2}=g_{3}=\cdots =0$ and the solution of the nonlinear Schrödinger equation is

(56) $$\psi =\rho_{0}\exp\left(i\theta \right)\frac{1+b\exp(\eta )}{1+\exp(\eta )}.$$

In order to obtain the two-soliton solution of the nonlinear Schrödinger equation, we start with solutions of (48) and (49) which depend on solutions of two simple equations for exponential functions

(57) $$f_{1}=\exp\left(\eta_{1}\right)+\exp\left(\eta_{2}\right);\;\;g_{1}=b_{1}\exp\left(\eta_{1}\right)+b_{2}\exp\left(\eta_{2}\right),$$

where $\eta_{i}=q_{i}x-\omega_{i}t+\sigma_{i}$, i=1,2. The substitution of (57) in (48) and (49) (Steps 6 and 7 of SEsM) leads to a system of algebraic equations which solution is

(58) $$\begin{array}{c} \omega_{i}=q_{i}\left(2k-\sqrt{4\rho_{0}^{2}-q_{i}^{2}};\;\;b_{i}=\exp\left(2i\phi_{i}\right)\right);\phi_{i}=\tan^{-1}-\left(\frac{q_{i}}{\omega_{i}-2kq_{i}}\right),\\ i=1,2. \end{array}$$

The substitution of (57), (58) in (50) and (51) leads to solutions containing single exponential function

(59) $$f_{2}=a_{12}\exp\left(\eta_{1}+\eta_{2}\right);\;\;g_{2}=b_{12}\exp\left(\eta_{1}+\eta_{2}\right),$$

where

$$a_{12}={\left(\frac{sin[\frac{1}{2}\left(\phi_{1}-\phi_{2}\right)]}{sin[\frac{1}{2}\left(\phi_{1}+\phi_{2}\right)]}\right)}^{2};\;\;b_{12}=b_{1}b_{2}a_{12},q_{i}=2\rho_{0}\sin\left(\phi_{i}\right),\;\;i=1,2.$$

The relationships for $f_{2}$ and $g_{2}$ leads to $f_{3}=f_{4}=\cdots =0$; $g_{3}=g_{4}=\cdots =0$ and the two-soliton solution of the nonlinear Schrödinger equation becomes

(60) $$\psi =\rho_{0}\exp\left(i\theta \right)\frac{1+\exp\left(\eta_{1}+2i\phi_{1}\right)+\exp\left(\eta_{2}+2i\phi_{2}\right)+a_{12}\exp\left(\eta_{1}+\eta_{2}+2i\phi_{i}+2i\phi_{2}\right)}{1+\exp\left(\eta_{1}\right)+\exp\left(\eta_{2}\right)+a_{12}\exp\left(\eta_{1}+\eta_{2}\right)}.$$

More complicated soliton solutions of the nonlinear Schrödinger equation can be obtained too. One has just to start by solution constructed by 3,4,… solutions of simple differential equations for the exponential function.

### 3.5. Example 3: The Soliton Solution of the Ishimori Equation

The last example is connected to the area of solid state physics: dynamics of spin chains. The Ishimori equation [100] is (2+1)-dimensional generalization of the Heisenberg ferromagnetic spin equation and it was introduced in order to explain the dynamics of a classical spin system on a plane. The Ishimori equation is

(61) $$\frac{\partial \vec{S}}{\partial t}=\vec{S}\wedge \left(\frac{\partial^{2}\vec{S}}{\partial x^{2}}+\sigma^{2}\frac{\partial^{2}\vec{S}}{\partial y^{2}}\right)+\frac{\partial \phi }{\partial y}\frac{\partial \vec{S}}{\partial x}+\frac{\partial \phi }{\partial x}\frac{\partial \vec{S}}{\partial y},$$

where

(62) $$\frac{\partial^{2}\phi }{\partial x^{2}}-\sigma^{2}\frac{\partial^{2}\phi }{\partial y^{2}}=-2\sigma^{2}\vec{S}\cdot \frac{\partial \vec{S}}{\partial x}\wedge \frac{\partial \vec{S}}{\partial y},$$

$\vec{S}\left(x,y,t\right)=\left(S_{1},S_{2},S_{3}\right)$ is the tree-dimensional spin unit vector, ϕ(x,y,t) is a scalar field, $\sigma^{2}=\pm 1$ and ∧ means *exterior* (*wedge*) product of corresponding vectors. Step 1 of SEsM is connected with the transformation of the nonlinearity of the studied equation [101,102]. Before Step 1 we introduce the stereographic projection of the spin of the unit sphere on a complex plane, the spin components can be written in terms of the stereographic variable ω ( $\omega \left(\vec{r},t\right)=\frac{S_{1}+iS_{2}}{1+S_{3}}$) as follows

(63) $$S^{+}=S_{1}+iS_{2}=\frac{2\omega }{1+|\omega |},\;\;\;S_{3}=\frac{1-|\omega {|}^{2}}{1+|\omega {|}^{2}}.$$

The Ishimori equation becomes

(64) $$i\frac{\partial \omega }{\partial t}+\frac{\partial^{2}\omega }{\partial x^{2}}+\sigma^{2}\frac{\partial^{2}\omega }{\partial y^{2}}-2\frac{\omega^{*}}{1+|\omega {|}^{2}}\left[{\left(\frac{\partial \omega }{\partial x}\right)}^{2}+\sigma^{2}{\left(\frac{\partial \omega }{\partial y}\right)}^{2}\right]-i\frac{\partial \phi }{\partial y}\frac{\partial \omega }{\partial x}-i\frac{\partial \phi }{\partial x}\frac{\partial \omega }{\partial y}=0,$$

where

(65) $$\frac{\partial^{2}\phi }{\partial x^{2}}-\sigma^{2}\frac{\partial^{2}\phi }{\partial y^{2}}=\frac{4i\sigma^{2}}{{\left(1+|\omega {|}^{2}\right)}^{2}}\left(\frac{\partial \omega^{*}}{\partial x}\frac{\partial \omega }{\partial y}-\frac{\partial \omega }{\partial x}\frac{\partial \omega^{*}}{\partial y}\right).$$

At Step 1 of SEsM, we perform the transformation

(66) $$\omega =\frac{g(x,y,t)}{f(x,y,t)},$$

where *f* and *g* are complex functions. This allows us to write the Ishimori equation in the bilinear form by means of the Hirota operators

(67) $$\begin{array}{ccc} & & \left(iD_{t}-D_{x}^{2}-\sigma^{2}D_{y}^{2}\right)\left(f^{*}\cdot g\right)=0\\ & & \left(iD_{t}-D_{x}^{2}-\sigma^{2}D_{y}^{2}\right)\left(f^{*}\cdot f-g^{*}\cdot g\right)=0\\ & & D_{x}\left[D_{x}\left(f^{*}f+g^{*}g\right)\cdot \left(f^{*}f+g^{*}g\right)\right]=-\sigma^{2}D_{y}\left[D_{y}\left(f^{*}f+g^{*}g\right)\right]\cdot \left(f^{*}f+g^{*}g\right)\\ & & \frac{\partial \phi }{\partial x}=-2i\sigma^{2}\frac{D_{y}\left(f^{*}\cdot f+g^{*}\cdot g\right)}{f^{*}\cdot f+g^{*}\cdot g}\\ & & \frac{\partial \phi }{\partial y}=-2i\sigma^{2}\frac{D_{x}\left(f^{*}\cdot f+g^{*}\cdot g\right)}{f^{*}\cdot f+g^{*}\cdot g} \end{array}$$

At Step 2 of SEsM, we use two expansions: one expansion for for *f* and one expansion for *g*:

(68) $$f=1+\sum_{n=1}^{\infty }\epsilon^{2n}f_{2n};\;\;\;g=\sum_{n=0}^{\infty }\epsilon^{2n+1}g_{2n+1}.$$

After substitution of (68) in (67) we arrive at a system of differential equations for the functions $f_{i}$ and $g_{i}$ and we have to solve these systems in a way analogous to the previous examples. In order to obtain the *N*-soliton solution we start by solution constructed by solutions of simple equations for exponential functions as follows

(69) $$g_{1}=\sum_{j=1}^{N}\exp\left(\xi_{j}\right),\;\;\;\xi_{j}=l_{j}x+m_{j}y+n_{j}t,$$

where $l_{j},m_{j},n_{j}$ are complex constants. We shall write some details about the single soliton solution of the Ishimori equation. We start from

(70) $$g_{1}=M\exp\left(\xi_{1}\right);\;\;\;f_{2}=exp\left[2\left(\xi_{1R}+\psi \right)\right],$$

where *M* is an arbitrary complex constant, $\xi_{1}=l_{1}x+m_{1}y+n_{1}t$, $n_{1}=i\left(l_{1}^{2}+\sigma^{2}m_{1}^{2}\right)$; $\xi_{1}$ has real and imaginary part: $\xi_{1}=\xi_{1R}+\xi_{1I}$ and $\exp(2\psi =\frac{\sigma^{2}m_{1}^{2}-l_{1}^{2}}{{\left(l_{1}+l_{1}^{*}\right)}^{2}-\sigma^{2}{\left(m_{1}+m_{1}^{*}\right)}^{2}})$. Let us consider further the case $\sigma^{2}=1$. Then we have to solve the system of equations arising by setting to 0 the relationships obtained for the different powers of ϵ. The solution is obtained as in the case of the previous two examples and the obtained results is

(71) $$\begin{array}{c} S^{+}=2E\frac{\left(l_{1R}^{2}m_{1R}+m_{1R}^{2}l_{1I}+L\right)\exp\left(i\xi_{1I}\right)\mathrm{sech}\left(\xi_{1R}\right)}{A+2B\tanh\left(\xi_{1R}\right)+C\tanh^{2}\left(\xi_{1R}\right)};\;\;\;S_{3}=1-\frac{2{\left(l_{1R}^{2}-m_{1R}^{2}\right)}^{3}{\mathrm{sech}}^{2}\left(\xi_{1R}\right)}{A+2B\tanh(\xi_{1R}+C\tanh^{2}\left(\xi_{1R}\right))}, \end{array}$$

where

(72) $$\begin{array}{ccc} A & = & 2l_{1R}^{6}-2m_{1R}^{2}\left(l_{1R}^{4}+l_{1R}^{3}m_{1I}\right)+m_{1R}^{4}l_{1I}^{2}+2l_{1R}^{2}l_{1I}m_{1R}^{3}+m_{1I}^{2}m_{1R}^{4}+3l_{1R}^{2}m_{1R}^{4}-m_{1R}^{6},\\ B & = & l_{1R}^{2}m_{1R}^{2}\left(m_{1R}^{2}+l_{1I}^{2}+m_{1I}^{2}+l_{1R}^{2}\right)+l_{1R}^{3}l_{1I}m_{1R}+l_{1I}\left(m_{1R}^{5}-l_{1R}^{5}\right)-l_{1R}m_{1I}m_{1R}^{4},\\ C & = & 2m_{1R}^{6}+2l_{1I}l_{1R}^{2}m_{1R}^{3}+l_{1R}^{4}l_{1I}^{2}+m_{1I}^{2}l_{1R}^{4}+3m_{1R}^{2}l_{1R}^{4}-2l_{1R}^{2}m_{1R}^{4}-2m_{1I}l_{1R}^{3}m_{1R}^{2}-l_{1R}^{6},\\ E & = & \frac{l_{1R}+im_{1R}}{l_{1R}^{2}-m_{1R}^{2}},\\ L & = & i\left(n_{1I}m_{1R}^{2}-l_{1R}^{3}\right)+\left[m_{1R}^{3}+l_{1I}l_{1R}^{2}+i\left(m_{iI}l_{1R}^{2}-l_{1R}m_{1R}^{2}\right)\right]. \end{array}$$

In a similar way we can obtain two-soliton solution, three-soliton solution, etc. of the Ishimori equation.

## 4. SEsM and Its Connection with the Inverse Scattering Transform Method

### 4.1. The Inverse Scattering Transform Method

Below we shall discuss the connection between SEsM and IST method for the case of Korteweg-de Vries equation. First of all we briefly remember application of the IST for the case of the KdV equation:

(73) $$\frac{\partial u}{\partial t}-6u\frac{\partial u}{\partial x}+\frac{\partial^{3}u}{\partial x^{3}}=0.$$

Researchers tried to solve this equation by means of some transformation. Almost 50 years ago Gardner, Greene, Kruskal, and Miura [48] tried the transformation

(74) $$u=\frac{1}{\psi }\frac{\partial^{2}\psi }{\partial x^{2}}+\lambda .$$

This can be written as

(75) $$\frac{\partial^{2}\psi }{\partial x^{2}}+\left(\lambda -u\right)\psi =0,$$

which is the Schrödinger equation where *u* depends on *x* and *t*. However, *t* can be treated as a parameter and then (75) will be considered as a problem for the scattering of a particle in a potential u(x,t) associated with the Korteweg-de Vries equation and described by the linear Schrödinger equation. If the time is a parameter, then for any value of *t*, there will be a separate scattering problem.

The substitution of (75) in (73) leads to

(76) $$\psi^{2}\frac{d\lambda }{dt}+\frac{\partial }{\partial x}\left(\psi \frac{\partial P}{\partial x}-\frac{\partial \psi }{\partial t}P\right)=0,$$

where

(77) $$P=\frac{\partial \psi }{\partial t}+\frac{\partial^{3}\psi }{\partial x^{3}}-3\left(\lambda +u\right)\frac{\partial \psi }{\partial x}.$$

We shall search for solutions of the Korteweg-de Vries equations that decay fast to 0 at ∣x∣→∞. The associated scattering problem described by (75) has two kinds of eigenvalues for λ: (i) For λ<0 the values of λ are discrete. We shall write for these eigenvalues $\lambda_{n}=-\kappa_{n}^{2}$ and for the corresponding eigenfunctions $\psi_{n}$ we have $|\psi_{n}|\to 0$ at ∣x∣→0 and $0<\int_{-\infty }^{\infty }dx\psi_{n}^{2}<\infty$. The integration of (76) from −∞ to *∞* leads to the conclusion that $\lambda_{n}$ do not depend on *t*; (ii) For λ>0 the number of possible values of λ (the spectrum of λ) is continuous and we shall assume that the values from this spectrum do not depend on *t*. Then $\frac{d\lambda }{dt}=0$ and from (76) and (77) we obtain

(78) $$P=\frac{\partial \psi }{\partial t}+\frac{\partial^{3}\psi }{\partial x^{3}}-3\left(\lambda +u\right)\frac{\partial \psi }{\partial x}=C\psi ,$$

where *C* does not depend on *x*. Then, (78) describes the evolution of ψ for a fixed value of the parameter λ in the case of continuous spectrum of values λ as well as for the case of discrete spectrum of values of λ.

Next we have to determine *C* which is done by considering (75) and setting $\lambda =\mu^{2}$. We consider functions which are proportional to exp(iμx), Imμ≥0 at x→+∞ and assume that the function of this kind is an asymptotic solution of (78) for any *t*. This leads to $C=-4i\mu^{3}$ and the system of equations becomes

(79) $$\frac{\partial^{2}\psi }{\partial x^{2}}+\left(\mu^{2}-u\right)\psi =0,$$

(80) $$\frac{\partial \psi }{\partial t}+\frac{\partial^{3}\psi }{\partial x^{3}}-3\left(\mu^{2}+u\right)\frac{\partial \psi }{\partial x}+4i\mu^{3}\psi =0.$$

The methodology which is used for calculation of u(x,t) comes from the inverse scattering problem from physics: the scattering potential *u* can be reconstructed on the basis of the knowledge of the scattering coefficient for the waves arriving from x=+∞ and on the basis of knowledge about the spectrum. This methodology has four steps. At step 1, we know u(x,0) and use this information to solve (79). This leads to discrete eigenvalues $\mu =i\kappa_{n}$ which correspond to the eigenfunctions $\psi_{n}$ and the scattering coefficient β of the incoming waves. The eigenfunctions are chosen as

(81) $$\psi_{n}\left(x\right)=\chi \left(i\kappa_{n},x\right).$$

χ have to satisfy the condition described above, namely, one works with functions that are proportional to exp(iμx), Imμ≥0 at x→+∞. In addition normalization coefficients

(82) $$\gamma_{n}=\frac{1}{\int_{-\infty }^{+\infty }dx\psi_{n}^{2}},$$

are introduced. At Step 2 one constructs the solution containing scattering part characterized by the scattering coefficient β. We assume that the solution ψ(k,x) of (79) has the following asymptotic behavior

(83) $$\begin{array}{ccc} \psi (k,x) & ∼ & \exp(-ikx)+\beta (k)\exp(ikx),\;\;x\to +\infty ,\\ \psi (k,x) & ∼ & \alpha (k)\exp(-ikx),\;\;x\to -\infty . \end{array}$$

*k* is a positive number, α is the transmission coefficient and β is the reflection coefficient. These coefficients are determined by substitution of the solution (83) in the (79). Thus, one can solve the scattering problem: finding $\kappa_{n}$, $\gamma_{n}$ and $\beta_{k}$ when the potential u(x,0) is known. At step 3 one has to determine the time behavior of the scattering parameters. It follows from (79) and (80) (remember that $\kappa_{n}$ do not change)

(84) $$\frac{d}{dt}\int_{-\infty }^{+\infty }dx\chi^{2}={\left[-2\chi \frac{\partial^{2}\chi }{\partial x^{2}}+4{\left(\frac{\partial \chi }{\partial x}\right)}^{2}+6\mu^{2}\chi^{2}\right]}_{-\infty }^{+\infty }-8i\mu^{3}\int_{-\infty }^{+\infty }dx\chi^{2}.$$

At x→±∞ one has $\mu =i\kappa_{n}$ and $\psi_{n}\left(x,t\right)=\chi \left(x,t,i\kappa_{n}\right)\to 0$. The normalization coefficient is

(85) $$c_{n}\left(t\right)=\gamma_{n}\exp\left(8\kappa_{n}^{3}t\right).$$

The solution of the scattering problem at x→∞ has the behavior

(86) ψ(k,x,t)∼f(k,t)exp(−ikx)+g(k,t)exp(ikx).

(86) must be an asymptotic solution of (80) when μ=k. This condition leads to the relationships $f\left(k,t\right)=\exp\left(-8ik^{3}t\right)$; g(k,t)=β and the reflection coefficient is

(87) $$b\left(k,t\right)=\frac{g(k,t)}{f(k,t)}=\beta \left(k\right)\exp\left(8ik^{3}t\right).$$

Finally, at step 4, one has to solve the inverse scattering problem. One has to find u(x,t) on the basis of known scattering data $\kappa_{n}$, $c_{n}\left(t\right)$ and b(u,t). This happens on the basis of the relationship

(88) $$u\left(x,t\right)=-2\frac{d}{dx}K\left(x,x,t\right),$$

where K(x,y,t) is the solution of a linear integral equation known as the equation of Gelfand–Levitan–Marchenko

(89) $$K\left(x,y,t\right)+B\left(x+y,t\right)+\int_{x}^{\infty }dzK\left(x,z,t\right)B\left(z+y,t\right)=0,$$

where

(90) $$\begin{array}{c} \begin{array}{c} B\left(x+y,t\right)=\sum c_{n}\left(t\right)\exp\left[-\kappa_{n}\left(x+y\right)\right]+\frac{1}{2\pi }\int_{-\infty }^{+\infty }dk\;\;b\left(k,t\right)\exp\left[ik\left(x+y\right)\right]=\\ \sum \gamma_{n}\exp\left[-\kappa_{n}\left(x+y\right)+8\kappa_{n}^{3}t\right]+\frac{1}{2\pi }\int_{-\infty }^{+\infty }dk\;\;\beta \left(k\right)\exp\left[ik\left(x+y\right)+8ik^{3}t\right]. \end{array} \end{array}$$

We note that the parameters $\kappa_{n}$, $\gamma_{n}$ and β(k) are determined on the basis of the knowledge of u(x,0) from step 1 of the schema.

### 4.2. Connection between SEsM and the Inverse Scattering Transform Method

We shall discuss below the connection between SEsM and IST for the case of Korteweg-de Vries equation. The main points are as follows. We skip Step 1 of SEsM and consider the particular case where no transformation of the solved equation is performed. Then we represent the searched solution as

(91) $$u\left(x,t\right)=\sum_{n=1}^{\infty }\epsilon^{n}u_{n}\left(x,t\right).$$

This is Step 2 of SEsM. One initially treats ϵ as a small parameter, obtains a solution for *u* and the powers of ϵ are absorbed in the corresponding parameters of the obtained solution. The introduction of (91) in the solved equation leads to a system of equations for $u_{n}$—Step 3 of SEsM. The obtained system of equations is solved on the basis of solutions constructed from solutions of simple equations for exponential functions (represented by a Fourier series constructed on the basis of these exponential functions. Note that Fourier series method for obtaining solutions of partial differential equations is a particular case of SEsM [74]). Then by means of the appropriate transformation the obtained solution for the case of small values of ϵ is transformed into a solution for finite values of ϵ.

Rosales [103] used the Fourier series in order to obtain the IST methodology for many equations. Below we shall use this excellent work in order to demonstrate the connection between SEsM and IST. We shall use the simple case of Burgers equation and then we shall discuss the methodology for the Korteweg-de Vries equation and in this case we shall arrive at the Gelfand–Levitan–Marchenko equation.

### 4.3. Example 1: The Burgers Equation

The Burgers equation is

(92) $$\frac{\partial u}{\partial t}+u\frac{\partial u}{\partial x}-\frac{\partial^{2}u}{\partial x^{2}}=0.$$

We skip Step 1 of SEsM: the transformation of nonlinearity and at Step 2 of SEsM we write the function u(x,t) by functions $u_{n}\left(x,t\right)$ as in (91). For now we shall treat ϵ as a “small” parameter. Our strategy is as follows: First we search for a solution for which $u_{n}\to 0$ when x→∞ and ϵ is small. Then we rewrite this solution to have a solution valid for all *x* and we shall no longer require ϵ to be small.

The equations for $u_{n}\left(x,t\right)$ can be obtained by substitution of (91) in (92) and collection of the terms containing equal powers of ϵ. We obtain

(93) $$\frac{\partial u_{n}}{\partial t}-\frac{\partial^{2}u_{n}}{\partial x^{2}}=-\sum_{j=1}^{n-1}u_{n-j}\frac{\partial u_{j}}{\partial x},n=2,3,\ldots$$

Now step by step we can solve the Equation (93) and we can obtain $u_{n}$. For n=1 we have

(94) $$\frac{\partial u_{1}}{\partial t}-\frac{\partial^{2}u_{1}}{\partial x^{2}}=0.$$

At Steps 3–5 of SEsM, we are going to represent $u_{n}$ by solutions of more simple equations. As in the case of the method of Hirota above, we shall use as simple equations the equations for exponential functions and we construct $u_{1}$ by solutions of these simple equations in the form of a Fourier transform

(95) $$u_{1}=\int_{C}d\lambda \left(k\right)\;\;\;\left(2ik\right)\exp\left[ikx-k^{2}t\right],$$

where dλ(k) is an appropriate measure in the complex plane C and the factor 2ik is introduced for convenience. The reason for the possibility of representation (95) is that (94) is a linear equation. We note that we can choose the measure above in such a way that $u_{1}$ is a superposition of a Fourier transform plus real exponentials

(96) $$u_{1}=\sum_{m}\alpha_{m}\exp\left[-\kappa_{m}x+\kappa_{m}^{2}\right]+\int_{-\infty }^{\infty }dk\;\;\beta \left(k\right)\left(2ik\right)\exp\left[ikx-k^{2}t\right],$$

where $\alpha_{m}$ are parameters and β(k) is a function.

The choice of solution for $u_{1}$ as (95) influences the equations for $u_{2}$, $u_{3}$, …. The equation for $u_{2}$ becomes

(97) $$\frac{\partial u_{2}}{\partial t}-\frac{\partial^{2}u_{2}}{\partial x^{2}}=-u_{1}\frac{\partial u_{1}}{\partial x}=\int_{C^{2}}d\lambda \left(k_{1}\right)d\lambda \left(k_{2}\right)\;\;\left(4ik_{1}^{2}k_{2}\right)\exp\left[i\left(k_{1}+k_{2}\right)x-\left(k_{1}^{2}+k_{2}^{2}\right)t\right],$$

and the equation for $u_{3}$ becomes

(98) $$\begin{array}{c} \frac{\partial u_{3}}{\partial t}-\frac{\partial^{2}u_{3}}{\partial x^{2}}=-u_{2}\frac{\partial u_{1}}{\partial x}-u_{1}\frac{\partial u_{2}}{\partial x}=\\ \int_{C^{3}}d\lambda \left(k_{1}\right)d\lambda \left(k_{2}\right)d\lambda \left(k_{3}\right)\;\;4i\left[k_{1}^{2}k_{2}+k_{1}k_{3}\left(k_{1}+k_{2}\right)\right]\exp\left\{i\left[\left(k_{1}+k_{2}+k_{3}\right)x+i\left(k_{1}^{2}+k_{2}^{2}+k_{3}^{2}\right)t\right]\right\}. \end{array}$$

We search for solution of (97) as

(99) $$u_{2}\left(x,t\right)=\int_{C^{2}}d\lambda \left(k_{1}\right)d\lambda \left(k_{2}\right)\;\;\Phi_{2}\left(k_{1},k_{2}\right)\exp\left[i\left(k_{1}+k_{2}\right)x-\left(k_{1}^{2}+k_{2}^{2}\right)t\right],$$

and for solution for $u_{3}$ we assume

(100) $$\begin{array}{c} u_{3}\left(x,t\right)=\int_{C^{3}}d\lambda \left(k_{1}\right)d\lambda \left(k_{2}\right)d\lambda \left(k_{3}\right)\;\;\Phi_{3}\left(k_{1},k_{2},k_{3}\right)\exp\left\{i\left[\left(k_{1}+k_{2}+k_{3}\right)x+i\left(k_{1}^{2}+k_{2}^{2}+k_{3}^{2}\right)t\right]\right\}. \end{array}$$

The substitution of (99) in (97) and of (100) in (98) leads to following relationships for $\Phi_{2}$ and $\Phi_{3}$

(101) $$\Phi_{2}\left(k_{1},k_{2}\right)=2ik_{1};\;\;\Phi_{3}\left(k_{1},k_{2},k_{3}\right)=2ik_{1}.$$

Next we assume the general form of the solution of the equations for $u_{n}$ to be:

(102) $$u_{n}\left(x,t\right)=\int_{C^{n}}{\left[d\lambda \left(k\right)\right]}^{n}\Phi_{n}\exp\left\{i\Omega_{n}\right\},$$

where ${\left[d\lambda \left(k\right)\right]}^{n}=d\lambda \left(k_{1}\right)\ldots d\lambda \left(k_{n}\right)$; $\Phi_{n}=\Phi_{n}\left(k_{1},\ldots ,k_{n}\right)$ and $\Omega_{n}=\left(k_{1}+\ldots +k_{n}\right)x+i\left(k_{1}^{2}+\ldots +k_{n}^{2}\right)t$. Because we have $\Phi_{2}=\Phi_{3}=2ik_{1}$ we assume further that

(103) $$\Phi_{n}\left(k_{1},\ldots ,k_{n}\right)=2ik_{1},$$

and this is true indeed as it can be seen by direct substitution of (102) in (93). Then we write the solution of Burgers equation at x→∞ and small values of ϵ

(104) $$u=\sum_{n=1}^{\infty }\epsilon^{n}\int_{C^{n}}{\left[d\lambda \left(k\right)\right]}^{n}\left(2ik_{1}\right)\exp\left\{i\Omega_{n}\right\}.$$

By means of the notation

(105) $$\zeta =\epsilon \int_{C}d\lambda \left(k\right)\exp\left\{ikx-k^{2}t\right\},$$

we can write *u* as

(106) $$u=\sum_{n=1}^{\infty }\epsilon^{n}\int_{C^{n}}{\left[d\lambda \left(k\right)\right]}^{n}\left(2ik_{1}\right)\exp\left\{i\Omega_{n}\right\}=\sum_{n=1}^{\infty }2\zeta^{n-1}\frac{\partial \zeta }{\partial x}.$$

We use the formula for summation of the power series

$$1+q+q^{2}+q^{k-1}+\ldots =\frac{1}{1-q},\;\;|q|<1,$$

and apply it to (106) with q=ζ. The result is

(107) $$u=2\frac{\partial \zeta }{\partial x}\frac{1}{1-\zeta }=-2\frac{\partial }{\partial x}\ln\left(1-\zeta \right).$$

We remember that ζ and thus 1−ζ where ϵ is a parameter, is the general solution of the heat equation. In addition (107) is exactly the Hopf-Cole transformation [46,47] which linearizes the Burgers equation and reduces it to the heat equation. Thus the obtained solution (106) is a solution of the Burgers equation not only for small ϵ and for x→∞ but also for large ϵ and for arbitrary *x*.

Let us now consider a more complicated equation: the Korteweg-de Vries equation.

### 4.4. Example 2: The Korteweg-de Vries Equation

We consider the Korteweg-de Vries equation

(108) $$\frac{\partial u}{\partial t}+6u\frac{\partial u}{\partial x}+\frac{\partial^{3}u}{\partial x^{3}}=0.$$

We skip Step 1 of SEsM (the transformation of the nonlinearity in (108)). At Step 2 of SEsM, we represent u(x,t) by means of other functions $u_{1},u_{2},\ldots$ which then will be connected to the solutions of the simple equations. We use the relationship (91) where ϵ is considered initially to be a small parameter. The substitution of (91) in (108) leads to equations for $u_{1},u_{2},\ldots$ as follows

(109) $$\frac{\partial u_{n}}{\partial t}+\frac{\partial^{3}u_{n}}{\partial x^{3}}=-6\frac{\partial }{\partial x}\sum_{j=1}^{n-1}u_{j}u_{n-j},n=2,3,\ldots$$

and the equation for $u_{1}$ is

(110) $$\frac{\partial u_{1}}{\partial t}+\frac{\partial^{3}u_{1}}{\partial x^{3}}=0$$

At Step 3 of SEsM, we shall use solutions of the obtained equations for $u_{1}$, $u_{2}$, … which are connected through Fourier series to the solution of a simple equation for exponential functions (Steps 4 and 5 of SEsM). The solution for $u_{1}$ is

(111) $$u_{1}=\int_{C}d\lambda \left(k\right)\;\left(-k\right)\exp\left[ikx+k^{3}t\right],$$

where dλ(k) is an appropriate measure in the complex plane C and the term (−k) is introduced for convenience. This choice of $u_{1}$ influences the form of equations for $u_{2}$, $u_{3}$, …. For an example the equations for $u_{2}$ and $u_{3}$ are

(112) $$\begin{array}{c} \frac{\partial u_{2}}{\partial t}+\frac{\partial^{3}u_{2}}{\partial x^{3}}=-6i\int_{C^{2}}d\lambda \left(k_{1}\right)d\lambda \left(k_{2}\right)\;\;k_{1}k_{2}\left(k_{1}+k_{2}\right)\exp\left\{i\left[\left(k_{1}+k_{2}\right)x+\left(k_{1}^{3}+k_{2}^{3}\right)t\right]\right\}, \end{array}$$

(113) $$\begin{array}{c} \frac{\partial u_{3}}{\partial t}+\frac{\partial^{3}u_{3}}{\partial x^{3}}=-6\frac{\partial }{\partial x}\left(u_{1}u_{2}+u_{2}u_{3}\right)=\\ =-6i\int_{C^{3}}d\lambda \left(k_{1}\right)d\lambda \left(k_{2}\right)d\lambda \left(k_{3}\right)\;\;\left(k_{1}+k_{2}\right)\left(k_{1}+k_{2}+k_{3}\right)\exp\left\{i\left[\left(k_{1}+k_{2}+k_{3}\right)x+\left(k_{1}^{3}+k_{2}^{3}+k_{3}^{3}\right)t\right]\right\}. \end{array}$$

The solutions for $u_{2},u_{3},\ldots$ are searched again in the form

(114) $$u_{n}\left(x,t\right)=\int_{C^{n}}{\left[d\lambda \left(k\right)\right]}^{n}\Phi_{n}\left(k_{1},\ldots ,k_{n}\right)\exp\left\{i\Omega_{n}\right\},$$

and by substitution of this relationship in equations for $u_{2},u_{3},\ldots$ we will determine $\Phi_{n}$ and $\Omega_{n}$ (Steps 6. and 7. of SEsM).

The substitution of (114) in (112) leads to

(115) $$\Phi_{2}\left(k_{1},k_{2}\right)=1;\;\;\Omega_{2}=\left(k_{1}+k_{2}\right)x+\left(k_{1}^{3}+k_{2}^{3}\right)t.$$

The substitution of (114) in (113) leads to

(116) $$\Phi_{3}\left(K_{1},k_{2},k_{3}\right)=-\frac{k_{1}+k_{2}+k_{3}}{\left(k_{1}+k_{2}\right)\left(k_{2}+k_{3}\right)};\;\;\Omega_{3}=\left(k_{1}+k_{2}+k_{3}\right)x+\left(k_{1}^{2}+k_{2}^{2}+k_{3}^{2}\right)t.$$

In addition we can obtain for the parameters of the solution $u_{4}$

(117) $$\Phi_{4}=\frac{k_{1}+k_{2}+k_{3}+k_{4}}{\left(k_{1}+k_{2}\right)\left(k_{2}+k_{3}\right)\left(k_{3}+k_{4}\right)};\;\;\Omega_{4}=\left(k_{1}+k_{2}+k_{3}+k_{4}\right)x+\left(k_{1}^{2}+k_{2}^{2}+k_{3}^{2}+k_{4}^{2}\right)t.$$

The continuation of the calculations leads us to

(118) $$u_{n}={\left(-1\right)}^{n+1}i\frac{\partial }{\partial x}\int_{C^{n}}{\left[d\lambda \left(k\right)\right]}^{n}\frac{\exp(i\Omega_{n})}{\prod_{j=1}^{n-1}\left(k_{j}+k_{j+1}\right)}.$$

The solution of the Korteweg-de Vries equation becomes

(119) $$u=i\frac{\partial }{\partial x}\left[\sum_{n=1}^{\infty }{\left(-\epsilon \right)}^{n}\int_{C^{n}}{\left[d\lambda \left(k\right)\right]}^{n}\frac{\exp(i\Omega_{n})}{\prod_{j=1}^{n-1}\left(k_{j}+k_{j+1}\right)}\right].$$

The straight summation of the infinite series in the sum in (119) is impossible because the different harmonics are coupled by the factors ${\left(k_{j}+k_{j+1}\right)}^{-1}$ but the summation still can be done as follows. We write (119) as

(120) $$u\left(x,t\right)=\frac{\partial }{\partial x}\sum_{n=1}^{n}{\left(-\epsilon \right)}^{n}\int_{C^{n}}d{\left[\lambda \left(k\right)\right]}^{n}\;\;\hat{p}\left(k_{1}\right)\hat{P}\left(k_{1},k_{2}\right)\hat{P}\left(k_{2},k_{3}\right),\ldots \hat{P}\left(k_{n-1},k_{n}\right)\hat{p}\left(k_{n}\right),$$

where

(121) $${\hat{p}}_{k}=\exp\left\{\frac{i}{2}\left[kx-\omega \left(k\right)t\right]\right\};\;\;\hat{P}\left(k,q\right)=i\frac{\hat{p}\left(k\right)\hat{p}\left(q\right)}{k+q},$$

and $\omega \left(k\right)=-k^{3}$. Now there are two possibilities for the measure dλ(k): to be discrete or to be continuous.

We consider first the case of discrete dλ(k). The integral from (120) can be replaced by a sum as follows

(122) $$\int_{C}d\lambda \left(k\right)f\left(k\right)=\sum_{m}a_{m}^{2}f\left(ik_{m}\right).$$

Then

(123) $$u=\frac{\partial }{\partial x}\sum_{n=1}^{\infty }{\left(-\epsilon \right)}^{n}\sum_{m_{j}}p_{m_{1}}P_{m_{1},m_{2}}P_{m_{2},m_{3}}\ldots P_{m_{n-1},m_{n}}p_{m_{n}},$$

where

(124) $$P_{mq}=\frac{p_{m}p_{q}}{k_{m}+k_{q}};\;\;p_{m}=a_{m}\hat{p}\left(ik_{m}\right)=a_{m}\exp\left\{\frac{1}{2}\left(-k_{m}x+k_{m}^{3}t\right)\right\},$$

(123) is a matrix product and if *p* is the column vector of all $p_{m}$ and *P* is the square matrix of all $P_{m}q$ we can write $p^{T}$ is the transpose of *p*

(125) $$u=-\epsilon \frac{\partial }{\partial x}\left[p^{T}{\left(I+\epsilon P\right)}^{-1}p\right].$$

For $k_{m}>0$ and real $a_{m}$ the matrix *P* is real symmetric and positive definite. Then *u* from (125) is nonsingular for ϵ>0 and ϵ can be absorbed in the coefficients $a_{m}$. This means that the solution (125) is no longer limited to small values of ϵ and we can set ϵ=1 (absorbing other values of ϵ in coefficients $a_{m}$).

We can write (125) in much more known form. In order to do this we observe that $\frac{\partial P}{\partial x}=-\frac{1}{2}pp^{T}$ and then

(126) $$u=2\frac{\partial }{\partial x}\mathrm{Tr}\left[{\left(I+P\right)}^{-1}\frac{\partial P}{\partial x}\right]=2\frac{\partial^{2}}{\partial x^{2}}\mathrm{Tr}\left[\ln\left(I+P\right)\right]=2\frac{\partial^{2}}{\partial x^{2}}\ln\det\left(I+P\right).$$

Thus we arrive at

$$u=2\frac{\partial^{2}}{\partial x^{2}}\ln\det\left(I+P\right),$$

which is the relationship for the multisoliton solution of the Korteweg-de Vries equation.

Up to now we have considered the case of discrete measure. In the case of continuous measure dλ(k) we arrive at the Gelfand–Levitan–Marchenko equation from the methodology of the IST as follows. We have to treat *P* as more general operator as square matrix. We write

(127) $$\left(Pf\right)\left(k\right)=\int_{C}d\lambda \left(l\right)\hat{P}\left(k,l\right)f\left(l\right),$$

and the solution of KdV equation becomes

(128) $$u=-\epsilon \frac{\partial }{\partial x}\int_{C}d\lambda \left(k\right)\left[\hat{p}{\left(k\right)[\left(I+\epsilon P\right)}^{-1}p\right]\left(k\right).$$

We can write the solution as

(129) $$u=2\frac{\partial }{\partial x}K\left(x,x\right),$$

where

(130) $$K\left(x,y\right)=-\frac{\epsilon }{2}p^{T}\left(x\right){\left[I+\epsilon P\left(x\right)\right]}^{-1}p\left(y\right).$$

After some calculation we obtain

(131) $$K\left(x,y\right)=-\frac{\epsilon }{2}p^{T}\left(x\right)p\left(y\right)-\frac{\epsilon }{2}\int_{x}^{\infty }dzK\left(x,z\right)p^{T}\left(z\right)p\left(y\right)$$

which is the GLM equation with

(132) $$B\left(x+y\right)=\frac{\epsilon }{2}p^{T}\left(x\right)p\left(y\right)=\frac{\epsilon }{2}\int d\lambda \left(k\right)\exp\left[i\left(\frac{k}{2}\left(x+y\right)+k^{3}t\right)\right].$$

Thus we have shown that SEsM is connected to the inverse scattering transform method for the Korteweg-de Vries equation. A similar connection can be proved also for other equations.

## 5. Several Particular Cases of SEsM and Their Connections with Other Methods

Below we show that many famous methods for obtaining particular exact solutions of nonlinear partial differential equations are connected to particular cases of SEsM. This is important to know in order to avoid errors when applying methods for obtaining exact solutions of nonlinear partial differential equations (for discussion see, e.g., [104,105]).

### 5.1. Homogeneous Balance Method and SEsM

The homogeneous balance method was discussed by Wang and et al. [106,107,108]. The method is as follows [107]. We consider the partial differential equation

(133) $$P(u,u_{x},u_{t},u_{xx},u_{xt},u_{tt},\ldots )=0,$$

where *P* is in general a polynomial function of its arguments, u=u(x,t), and the subscripts denote the partial derivatives. A function w=w(x,t) is called a quasisolution of Equation (133), if there exists a function f=f(w) of a single variable so that a suitable linear combination of the following functions

(134) $$f\left(w\right),{\left[f\left(w\right)\right]}_{x},{\left[f\left(w\right)\right]}_{t},{\left[f\left(w\right)\right]}_{xx},{\left[f\left(w\right)\right]}_{xt},{\left[f\left(w\right)\right]}_{tt},\ldots$$

is actually a solution of Equation (133). Four steps are needed to find f(w). At step 1, one chooses a suitable linear combination of the functions from (134). The coefficients in this linear combination must be determined, so that the highest nonlinear terms and the highest order partial derivative terms in the given equation are both transformed into the polynomials with a highest equality degree in partial derivatives of w(x,t) in spite of f(w) and its various derivatives. These equal highest degrees determine the form of the linear combination. At step 2, after a substitution of the linear combination chosen in the first step into Equation (133), followed by a collection of all terms with the highest degree of derivatives of *w*(*x*,*t*) and setting its coefficient to zero, one obtains an ordinary differential equation for f(w) and then one solves it. At step 3, starting from the ODE and its solution obtained above, the nonlinear terms of various derivatives of f(w) in the relationship obtained in the second step can be replaced by the corresponding higher order derivatives of f(w). Then by a collection of all terms with the same order derivatives of f(w) and by setting the coefficient of each order derivative of f(w) to zero respectively, one obtains a set of equations for w(x,t). The left hand sides of these equations are the *k* degree homogeneous functions in various derivatives of w(x,t), where *k* is the order of $f^{\left(k\right)}$. In view of the homogeneous property of these equations one can expect that w(x,t) is an exponential function with some constants to be determined. Substituting the exponential function assumed into each *k* degree homogeneous equation in partial derivatives of w(x,t), one obtains a set of nonlinear algebraic equations for some constants to be determined. If there exists a solution for these nonlinear algebraic equations, then w(x,t) and the coefficients of the linear combination chosen in the first step can be determined. At step 4, by substitution of f(w), w(x,t), and some constants obtained in the second and third steps into the combination chosen in the first step, and after doing some calculations, one obtains an exact solution of Equation (133).

Now we show that the homogeneous balance method is connected to a particular case of SEsM.

**Assumption** **2.** *The homogeneous balance method is connected to the particular case of SEsM where there is no transformation of the nonlinearity of the equation (Step 1 of SEsM is skipped) and the function f(w) is the solution of a single simple equation (i.e., we have just one function f(w) at Step 2 of SEsM and the form of the function F at Step 2 of SEsM is a linear combination of the functions from (134)). The used simple equation is the equation for the function f(w) at Steps 3, 4, and 5 of SEsM. The balance in the homogeneous balance method is a particular case of the balance procedure from Step 6 of SEsM and the algebraic system is the same as the algebraic system from Step 7 of SEsM.*

We start from SEsM and then impose restrictions in order to come to the particular case connected to the homogeneous balance method. Let us consider the partial differential equation (133). We skip Step 1 of SEsM (no transformation of the nonlinearity of the equation (133)) and come to the particular class of SEsM methodology without transformation of the nonlinearity of the solved equation. At Step 2 of SEsM, we take the particular case of a single function *F* which has particular form to be a linear combination of the functions from (134). Note that the functions from (134) depend on a single function *w*. This is another restriction on the function *F* from Step 2 of SEsM. At Step 3 of SEsM we make traveling-wave ansatz and thus we consider the particular case of SEsM when the solutions are travelling waves. We skip Step 4 of SEsM. The simple equation from Step 5 of SEsM is a particular case: this equation is chosen to be the differential equation for f(w) from the homogeneous balance method. The balance in the homogeneous balance method is a particular case of the balance procedure at Step 6 of SEsM for the case of balance of the highest powers in relationship containing monomials which are made of functions of a single variable. The algebraic system of the homogeneous balance method is the algebraic system from Step 7 of SEsM. Thus we have started from the general SEsM and by applying restrictions on it, we arrived at one particular case of SEsM connected to the homogeneous balance method.

Now let consider the homogeneous balance method in the version of Fan and Zhang [109]. They consider the equation (133) and search for a traveling wave solutions u(x,t)=u(ξ)=u(x−λt) of this equation. The solution is searched in the form

(135) $$u\left(\xi \right)=\sum_{i=0}^{m}a_{i}v^{i}\left(\xi \right),$$

where

(136) $$\frac{dv}{d\xi }=k\left(1-v^{2}\right),$$

and *k* and λ are parameters. The solution of (136) is

(137) v=tanh(kξ);v=coth(kξ).

Now let us show that this version of the homogeneous balance method is connected to a particular case of SEsM.

**Assumption** **3.** *The homogeneous balance method in the version of Fan and Zhang is connected to a particular case of SEsM where, there is no transformation of the nonlinearity of the equation (Step 1 of SEsM is skipped), just one function u is used and the particular form (135) is used to relate the function u to the solution of the simple equation for the function v-Equation (136).*

We start from SEsM and impose restrictions on it in order to reduce SEsM to the particular case connected to the homogeneous balance method in the version of Fan and Zhang. First, we consider the particular case of SEsM where no transformation of the nonlinearity of the solved equation is made. At Step 2 of SEsM, we consider again a particular case where a single function *u* is used. At Step 3 of SEsM we consider the particular case where we search for a traveling wave solution of the solved equation. At Step 4 of SEsM we assume a particular case of the relationship between the functions *u* and *v*, namely (135). At Step 5 of SEsM we again consider the particular case where the simple equation for *v* is (136). Steps 6 and 7 of SEsM follow and we may obtain an exact traveling wave solution of the solved equation. Thus we have started from SEsM and by considering particular cases of this methodology we have reduced it to a particular case connected to the homogeneous balance method in the version of Fan and Zhang.

### 5.2. Extended Homogeneous Balance Method and SEsM

The extended homogeneous balance method [110] extends the Fan and Zhang version of the homogeneous balance method by use of two simple equations of the same kind and of two traveling wave variables. Below we consider an assumption for this version of the homogeneous balance method and then we consider a version of the homogeneous balance method, which is based on an arbitrary number of simple equations and for any of these equations there is a separate traveling wave coordinate.

**Assumption** **4.** *The extended homogeneous balance method in the version of El-Wakil et al. [110] is connected to particular case of SEsM for the case when there is no transformation of the nonlinearity of the equation (Step 1 of SEsM is skipped), two functions u and v is used and for these functions particular form (135) is used to relate them to the solution of two simple equations of the same kind*

(138) $$\frac{d\phi }{d\xi_{1,2}}=a_{1,2}\phi^{2}+c_{1,2}$$

*where $a_{1,2}$ and $c_{1,2}$ are parameters and $\xi_{1,2}=\alpha_{1,2}x+\beta_{1,2}t$ are two traveling wave coordinates.*

We consider SEsM and impose restrictions on it in order to reduce SEsM to the particular case connected to the homogeneous balance method in the version of El-Wakil et al. We consider the particular case of SEsM where no transformation of the nonlinearity of the solved equation is made. At Step 2 of SEsM, we consider another particular case where two functions *u* and *v* will be used. At Step 3 of SEsM we consider the particular case where we search for a traveling wave solution of the solved equation. At Step 4 of SEsM we assume a particular case of the relationship between the functions u,v and ϕ, namely (135). At Step 5 of SEsM we again consider the particular case where the simple equations for ϕ are of the kind (138). Steps 6 and 7 of SEsM follow and we may obtain an exact traveling wave solution of the solved equation. Thus we have started from SEsM and by means of considering particular cases of this methodology we have reduced it to a particular case connected to the Extended homogeneous balance method in the version of El Wakil et al.

Now we are going to consider the much more extended homogeneous balance method (MMEHBM) and to show that this method is connected to a particular case of SEsM. In the MMEHBM we do not use the transformation of the nonlinearity of the solved equation. We use arbitrary number of functions $u_{1},u_{2},\ldots$. Any of these functions is represented by a power series of a function of corresponding traveling wave coordinate $\xi_{1},\xi_{2},\ldots$ and for the last function there is a separate simple equation (all of these simple equations can be different).

**Assumption** **5.** *The MMEHBM is connected to a particular case of SEsM where there is no transformation of the nonlinearity of the equation (Step 1 of SEsM is skipped), arbitrary number of functions $u_{1},u_{2},\ldots$ are used, any of these functions is represented by a power series of a function of corresponding traveling wave coordinate $\xi_{1},\xi_{2},\ldots$ and for the last function there is a separate simple equation (all of these simple equations can be different).*

In general, MMEHBM will be used for obtaining exact traveling wave solutions of systems of nonlinear partial differential equations. We consider SEsM and impose restrictions on it in order to reduce SEsM to the particular case connected to MMEHBM. First, we consider the particular case of SEsM where no transformation of the nonlinearity of the solved equation is made at Step 1 of SEsM. At Step 2 of SEsM, we consider again a particular case where we use one function $u_{i}$, (i=1,2,…) for any of the *i*-th solved equations. At Step 3 of SEsM, we consider the particular case where we search for a traveling wave solution of the solved equation and the traveling waves associated with the different functions $u_{i}$ can have different traveling wave coordinates. At Step 4 of SEsM, we assume a particular case of the relationship between the functions $u_{i}$ and $\phi_{i}$, which is of the kind (135). We note that this is a large restriction on SEsM. At Step 5 of SEsM, we again consider separate simple equation for $\phi_{i}\left(\xi_{i}\right)$ which is of the kind (138). Steps 6 and 7 of SEsM follow and we may obtain an exact traveling wave solution of the solved equation. Thus we have started from SEsM and by means of considering particular cases of this methodology we have reduced it to the particular case connected to MMEHBM.

### 5.3. Auxiliary Equation Method and SEsM

The summary of the auxiliary equation method is as follows [111]. One considers nonlinear partial differential equation for u(x,t) in the form

(139) $$H(u,u_{x},u_{t},u_{xx},u_{xt},u_{tt},\ldots )=0.$$

Then traveling wave variable ξ=x−ωt is introduced and Equation (139) becomes

(140) $$G(u,u_{\xi },u_{\xi \xi },\ldots )=0.$$

The solution of (140) is searched as

(141) $$u\left(\xi \right)=\sum_{i=0}^{n}a_{i}z^{i}\left(\xi \right),$$

where $a_{i}$ are parameters and z(ξ) is the solution of the auxiliary equation

(142) $${\left(\frac{dz}{d\xi }\right)}^{2}=az{\left(\xi \right)}^{2}+bz{\left(\xi \right)}^{3}+cz{\left(\xi \right)}^{4}.$$

Equations (141) and (142) are introduced in Equation (140) and then for the obtained relationship, one equates to 0 coefficients of the powers of z(ξ). The solution of the obtained system of algebraic equations leads to solution of solved equation (139).

Now we show that the auxiliary equation method is connected to a particular case of SEsM.

**Assumption** **6.** *The auxiliary equation method is connected to a particular case of SEsM where there is no transformation of the nonlinearity of the equation (Step 1 of SEsM is skipped); Function F at Step 2 of SEsM has particular form—(141)); just one simple equation is used that this simple equation has the form (142) which is a particular case of the form which can be used in SEsM.*

We start from SEsM, impose restrictions on it and reduce SEsM to the particular case connected to the auxiliary equation method. At Step 1 of SEsM we do not transform the nonlinearity of the solved equation (we skip this step). At Step 2 of SEsM we use one of the possible forms of the function *F*—(141). We skip Steps 3 and 4 of SEsM as the function z(ξ) in the auxiliary equation method is directly connected to the solution of the used simple equation. Then we consider the particular case of SEsM when only 1 simple equation is used and we restrict further SEsM by the assumption that the simple equation is of the form (142). In such a way we reduce SEsM to a particular case connected to the auxiliary equation method.

Now let us show that a generalized version of the auxiliary equation method is connected to a particular case of SEsM. The generalization is in two directions. First, we can use many relationships for *F* instead of (141) and second we can use many relationships for the simple equations instead of (142). We call this generalized version of the auxiliary equation method GAEM—general auxiliary equation method. GAEM is formulated as follows. One considers nonlinear partial differential equation for u(x,t) in the form

(143) $$H(u,u_{x},u_{t},u_{xx},u_{xt},u_{tt},\ldots )=0.$$

Then, traveling wave variable ξ=x−ωt is introduced and Equation (139) becomes

(144) $$G(u,u_{\xi },u_{\xi \xi },\ldots )=0.$$

The solution of (140) is searched in any form and not only in the form (141). Equation (141) is just a particular case of the possible forms that can be used in GAEM. z(ξ) can be solution of an arbitrary auxiliary equation. (142) is just one possibility for an auxiliary equation which can be used in GAEM. The introduction of the assumed form of the solution and of the assumed form of the auxiliary equation in (143) leads to a relationship consisting of coefficients multiplied by functions. We set to zero these coefficients and obtain a system of nonlinear algebraic equations. Any nontrivial solution of this system leads to an exact solution of (143).

Now we show that GAEM is connected to a particular case of SEsM.

**Assumption** **7.** *The general auxiliary equation method (GAEM) is connected to a particular case of SEsM where there is no transformation of the nonlinearity of the equation (Step 1 of SEsM is skipped); Function F at Step 2 of SEsM has particular form and this is the corresponding form used by GAEM; just one simple equation is used that this simple equation has the particular form which is the corresponding form used by GAEM.*

We start from SEsM, impose restrictions on it and reduce SEsM to a particular case connected to GAEM. At Step 1 of SEsM we do not transform the nonlinearity of the solved equation (we skip this step). At Step 2 of SEsM we use a possible form of the function *F*—the corresponding form used when GAEM is applied. This possible form is just one of the many forms that can be used in SEsM. We skip Steps 3 and 4 of SEsM as the function z(ξ) in the auxiliary equation method is directly connected to the solution of the used simple equation. Further, we consider the particular case of SEsM when only 1 simple equation is used and we restrict further SEsM by the assumption that this simple equation is of the form used by GAEM. In such a way we reduce SEsM to a particular case connected to the general auxiliary equation method.

An illustration of a particular case of GAEM can be seen in [112].

### 5.4. Jacobi Elliptic Function Expansion Method, F-Expansion Method and SESM

We show first that the Jacobi elliptic function expansion method (JEFEM) is connected to a particular case of SEsM. Then we describe general Jacobi elliptic function expansion method (GJEFEM) and show that it is connected to a particular case of SEsM. Finally, we list several methods used in the literature which are particular cases of GJEFEM.

The classic form of JEFEM is as given by [113]. One considers the following nonlinear partial differential equation for u(x,t)

(145) $$N(u,u_{x},u_{t},u_{xx},u_{xt},u_{tt},\ldots )=0,$$

and searches for traveling wave solutions in the form

(146) u=u(ξ):ξ=k(x−ct),

where *k* and *c* are parameters. u(ξ) is searched in the form of power series of the Jacobi elliptic function sn(ξ,m) where *m* is the modulus of the function sn,

(147) $$u\left(\xi \right)=\sum_{j=0}^{n}a_{j}\mathrm{sn}{\left(j,m\right)}^{j}.$$

This is a generalization of the tanh-method because for m=1sn(ξ,1)=tanh(ξ). The substitution of (146) and (147) in (145) can lead to a system of nonlinear algebraic equations and any nontrivial solution of this system leads to an exact traveling wave solution of the solved Equation (145).

**Assumption** **8.** *The Jacobi elliptic function expansion method (JEFEM) is connected to a particular case of SEsM where there is no transformation of the nonlinearity of the equation (Step 1 of SEsM is skipped); Function F at Step 2 of SEsM has particular form—(147)); just one simple equation is used that this simple equation is the differential equation for the elliptic function sn.*

We start from SEsM, impose restrictions on it and reduce SEsM to the particular case connected to JEFEM. At Step 1 of SEsM we do not transform the nonlinearity of the solved equation (we skip this step). Additional restriction is that we search for a traveling wave solution of the solved equation (145). At Step 2 of SEsM we use a possible form of the function *F*—(147). This form is just one of the many forms that can be used in SEsM. We skip Steps 3 and 4 of SEsM as the function from (147) in the JEFEM is directly connected to the solution of the used simple equation which is the equation for the Jacobi elliptic function sn. The use of only one simple equation is a further restriction on SEsM. By means of all restrictions above, we reduce SEsM to the particular case connected to JEFEM.

Next we formulate general Jacobi elliptic function expansion method (GJEFEM). By this method we solve in general a system of *N* nonlinear partial differential equations and search for traveling wave solutions based on different coordinates $\xi_{i}=\alpha_{i}x-\beta_{i}t$, i=1,2,…,N. The solution is searched as function

(148) $$u_{i}\left(\xi_{1},\ldots ,\xi_{n}\right)=U_{i}\left[h_{1}\left(\xi_{1}\right),\ldots ,h_{N}\left(\xi_{N}\right)\right]$$

of the functions $f_{1},\ldots ,f_{N}$ and each of these functions is a solution of a differential equation for the Jacobi elliptic functions

(149) $${\left(\frac{df_{i}}{d\xi_{i}}\right)}^{2}=a_{i}f_{i}^{4}+b_{i}f_{i}^{2}+c_{i}.$$

We show below that the GJEFEM is connected to a particular case of SEsM.

**Assumption** **9.** *The general Jacobi elliptic function expansion method is connected to a particular case of SEsM where, there is no transformation of the nonlinearity of the equation (Step 1 of SEsM is skipped); Functions $u_{i}$ at Step 2 of SEsM have particular form—(148)); and the simple equations are of the kind of the differential equation for the Jacobi elliptic functions (149).*

We start from SEsM, impose restrictions on it and reduce SEsM to JEFEM. At Step 1. of SEsM we do not transform the nonlinearity of the solved equation (we skip this step). An additional restriction is that we search for a traveling wave solution of the solved Equation (145). At Step 2 of SEsM we use a possible form of the functions $u_{i}$—(148). We skip Steps 3 and 4 of SEsM as the functions from (148) in the JEFEM are directly connected to the solution of the used simple equations which are of the kind of the differential equation for the Jacobi elliptic functions (149). This is an additional restriction on SEsM. By means of all restrictions above, we reduce SEsM to a particular case connected to GJEFEM.

Below, we list several particular cases of GJEFEM.

1. JEFEM is a particular case of GJEFEM for the case of just one solved nonlinear partial differential equation and when the simple equation is the equation for the Jacobi elliptic function sn and in addition the function *U* is a power series of the function sn.
2. Parks et al. [114] and Fu et al. [115] use expansions based on the elliptic functions cn, dn and cs. This is a particular case of GJEFEM when one simple equation is used and this simple equation is of the kind of (149).
3. Fan and Zhang [116] present an interesting application which is an extension of JEFEM for the case of two functions $u_{1,2}$ and single simple equation and by means of this extension they obtain solutions of the coupled Schrödinger-KdV system and of two-dimensional Davey–Stewartson equation. This extension of JEFEM is a particular case of GJEFEM when two functions $u_{1,2}$ are used with the same argument and when the simple equation is the differential equation for the elliptic function sn.
4. Another particular case of GJEFEM is applied by Yan [117] who treated a (2 + 1)-dimensional integrable Davey–Stewartson-type equation for the case of 2 spatial coordinates and travelling wave solutions. We note that SEsM allows treating equations with more that one spatial coordinate and the travelling waves can travel with different velocities which is a more general case than the case discussed by Yan where we have a single traveling wave despite the two spatial coordinates presented. Yan uses the following form of the function $u_{i}$, i=1,2,3
  (150) $$u_{i}\left(\xi \right)=a_{i0}+\sum_{j=1}^{n}f_{k}^{j-1}\left(\xi \right)\left[a_{ij}f_{k}\left(\xi \right)+b_{ij}g_{k}\left(\xi \right)\right],$$
  where $f_{k}$ and $g_{k}$, k=1,…,12 are Jacobi elliptic functions (i.e., are functions which satisfy the simple equation of the kind (149)). (150) is a particular form of the function $U_{i}$ from GJEFEM and the simple equations are equations for Jacobi elliptic functions as in GJEFEM.
5. Another particular case of GJEFEM is used in [118]. The simple equations used there are for Jacobi elliptic functions and the particular case of the used single function *U* is
  (151) $$U=a_{0}+\sum_{i=1}^{N}{\mathrm{sn}}^{-1}\left(\xi ,m\right)\left[a_{i}\mathrm{sn}\left(\xi ,m\right)+b_{i}\mathrm{cn}\left(\xi ,m\right)\right].$$
6. Liu and Fan [119] apply particular case of GJEFEM for the case of two spatial coordinates and time. These three variables are combined to produce a single traveling wave coordinate which allows the use of single variable simple equations. Wang et al. [120] use also a particular case of GJEFEM for the case of two spatial variables and time and combine all these variables in a single traveling wave variable. The new point in this article is the particular form of the functions $U_{i}$
  (152) $$U_{i}=a_{i0}+\sum_{j=1}^{m_{1}}\left[a_{ij}\frac{{\mathrm{sn}}^{j}\left(\xi ,m\right)}{{\left(\mu \mathrm{sn}\left(\xi ,m\right)+1\right)}^{j}}+b_{ij}\frac{{\mathrm{sn}}^{j-1}\left(\xi ,m\right)\mathrm{cn}\left(\xi ,m\right)}{{\left(\mu \mathrm{sn}\left(\xi ,m\right)+1\right)}^{j}}\right].$$
7. Ye at al. [121] extend (152) and use the following particular case for the functions $U_{i}$
  (153) $$U_{i}=a_{i0}+\sum_{j=1}^{m_{1}}\left[\frac{{a_{i,2j-1}\mathrm{sn}}^{j}\left(\xi ,m\right)}{{\left(\mu \mathrm{sn}\left(\xi ,m\right)+\mu_{2}\mathrm{cn}\left(\xi ,m\right)+1\right)}^{j}}+\frac{a_{i,2j}{\mathrm{sn}}^{j-1}\left(\xi ,m\right)\mathrm{cn}\left(\xi ,m\right)}{{\left(\mu \mathrm{sn}\left(\xi ,m\right)+\mu_{2}\mathrm{cn}\left(\xi ,m\right)+1\right)}^{j}}\right].$$
  Other variants for $U_{i}$ are proposed by Wang et al. [122], Chen and Wang [123], Lü [124], Abdou and Elhanbaly [125], El-Sabbagh and Ali [126,127].
8. Another particular case of GJEFEM is the F-expansion method which have the same ideology as JEFEM but only the form of the simple equations for the Jacobi elliptic functions are not specified. In the different variants of the F- expansion method one uses different particular cases for the functions $U_{i}$ from GJEFEM [128,129,130,131].

### 5.5. Modified Simple Equation Method and SEsM

According to [132] the modified simple equation method is as follows. One considers the nonlinear partial differential equation which can be reduced to an ordinary partial differential equation for the function u(z)

(154) $$P(u,u_{z},u_{zz},u_{zzz},\ldots )=0$$

(154) is solved by means of the ansatz

(155) $$u\left(z\right)=\sum_{k=0}^{N}A_{k}{\left(\frac{\psi_{z}}{\psi }\right)}^{k}.$$

Above $A_{k}$ are constants and $A_{N}\neq 0$. The function ψ is a solution of some ordinary differential equation of a lesser order than (154) (called simplest equation) and solutions of these simplest equations are known. One uses the finite series (155) in order to represent the solution *u* through the solution of the simplest equation. In doing this, one has to determine the value of *N* by means of the balance of power of the leading terms of the relationship which is obtained after the substitution of (155) in (154). This relationship is polynomial of $\frac{\psi_{z}}{\psi }$ and by setting to 0 of the coefficients to the powers of $\frac{\psi_{z}}{\psi }$ one obtains a system of nonlinear algebraic equations which solution leads to an exact solution of (154).

Now we show that the modified simple equation method is connected to a particular case of SEsM.

**Assumption** **10.** *The modified simple equation method is connected to a particular case of SEsM where there is no transformation of the nonlinearity of the equation (Step 1 of SEsM is skipped); Function F at Step 2 of SEsM has particular form—(155)) and just one simple equation is used.*

We start from SEsM, impose restrictions on it and reduce SEsM to a particular case connected to the Modified Method of Simple Equation. At Step 1 of SEsM we do not transform the nonlinearity of the solved equation (we skip this step). An additional restriction is that we search for solution of the solved equation which depends on a single coordinate *z*—(154). At Step 2 of SEsM we use the form (155) of the function *F*— this form is just one of the many forms that can be used in SEsM. We skip Steps 3 and 4 of SEsM as the function from (155) in the JEFEM is directly connected to the solution of the used simple equation which in this case is called the simplest equation. The use of only one simple equation is a further restriction on SEsM. By means of all restrictions above, we reduce SEsM to a particular case connected to the Modified Method of Simple Equation.

### 5.6. Trial Function Method and SEsM

The trial function method is described in [133,134] and it is as follows. We consider a nonlinear partial differential equation

(156) $$N(u,u_{x},u_{t},u_{xx},u_{xt},u_{tt},\ldots )=0,$$

and take a trial function y(x,t) in order to construct a solution u(y) of (156). The we substitute u(y) in (156) and determine the parameters of the solution.

**Assumption** **11.** *The trial function method is connected to a particular case of SEsM where there is no transformation of the nonlinearity of the equation (Step 1 of SEsM is skipped); Function F at Step 2 of SEsM has particular form—u(y), where y (the trial function) is the solution of a single simple equation.*

We start from SEsM, impose restrictions on it and reduce SEsM to a particular case connected to the trial function method. At Step 1 of SEsM we do not transform the nonlinearity of the solved equation (we skip this step). An additional restriction is that we search for a solution of the solved equation which depends on a single coordinate which can be a traveling wave coordinate or another kind of coordinate. At Step 2 of SEsM we use a particular form of the function *F* which is constructed by means of trial function. In most cases, *F* is presented by a finite power series of the trial function. The trial function is a solution to one simple equation. Thus, by means of the restrictions above, we reduce SEsM to a particular case connected to the trial function method.

### 5.7. First Integral Method and SEsM

The first integral method for obtaining exact solutions of nonlinear partial differential equations is as follows [135]. One wants to obtain exact solutions of the nonlinear partial differential equation

(157) $$P(u,u_{x},u_{t},u_{xx},u_{xt},u_{tt},\ldots )=0.$$

In order to do this one converts (157) to an ordinary differential equation by the traveling wave ansatz u(x,t)=U(z)=u(kx−ωt). Then one introduces X=U and $Y=U_{z}$ and writes (157) as system of equations

(158) $$Y=X_{z}$$

(159) $$Y_{z}=F\left(X,Y\right).$$

The solution is obtained by the assumption that the derivative of the relationship $Q\left(X,Y\right)=\sum_{i=0}^{m}a_{i}\left(X\right)Y^{i}$ can be represented as

(160) $$\frac{dQ}{dz}=\left[g\left(X\right)+h\left(X\right)Y\right]\sum_{i=0}^{m}a_{i}\left(X\right)Y^{i}$$

which together with (159) allows computation of the solution.

**Assumption** **12.** *The first integral method is connected to a particular case of SEsM for the case when equations of the kind*

(161) $$X_{zz}=F\left(X,X_{z}\right)$$

*are considered, there is no transformation of the nonlinearity of the equation (Step 1 of SEsM is skipped); one simplest equation is used and this simplest equation is determined by the condition (160).*

We stress that the first integral method can be applied to the restricted class of equations (161). This restricted class is obtained from (159) by substitution of (158) there. We start from SEsM, impose restrictions on it and reduce SEsM to the trial function method. At Step 1 of SEsM we do not transform the nonlinearity of the solved equation (we skip this step). An additional restriction is that we search for a solution of the solved equation that depends on a single coordinate which can be a traveling wave coordinate or another kind of coordinate. (160) imposes a further restriction on *X* and plays the role of the implicit simple equation which together with (161) determine the solution of (157). In this process one has to use a polynomial form of $a_{i}\left(X\right)$ and to determine the coefficients of these polynomials similar to Steps 6 and 7 of SEsM. Thus first integral method is connected to a particular case of SEsM for obtaining solutions for the limited class of equations (159) under the assumption that (160) holds.

## 6. Concluding Remarks

In this text, we present the Simple Equations Method (SEsM) for obtaining exact solutions of nonlinear differential equations and discuss its connections with other methods for obtaining exact solutions of nonlinear differential equations. Special importance is given to the connection to two of the most famous method for obtaining exact solutions of integrable nonlinear partial differential equations - method of Hirota and the inverse scattering transform method. We show that one particular case of SEsM is connected to the Hirota method. This means that the SEsM is a useful tool for obtaining exact solutions of nonlinear integrable differential equations and can lead to multisoliton solutions of such equations. Then we show that SEsM can be connected to the famous inverse scattering transform method. This is done in the case of the Korteweg-de Vries equation and much more research on this connection will be presented in the future. In addition to solutions of integrable nonlinear differential equations SEsM can lead also to exact particular solutions of nonintegrable nonlinear differential equations. Actually, the simplest version of SEsM (called Modified Method of Simplest Equation and based on representation of the searched solution as power series of the solution of single simple equation [85,86]) was intended exactly to solve the problem of obtaining particular exact solutions of nonintegrable nonlinear differential equations. Finally, we show that many other methods for obtaining particular exact solutions of nonlinear nonintegrable differential equations are connected to particular cases of SEsM. Many of these particular cases are discussed above in the text. Some more cases are listed in [74]. We note that additional discussion of other particular cases of the SEsM will be presented elsewhere and this is connected to many important questions about the methodology for obtaining exact solutions of nonlinear differential equations raised in [104,105].

We believe that the SEsM algorithm is useful one and it presents a fruitful way for obtaining solutions of nonlinear differential equations. The development of SEsM is far from finished. More research on the possible classes of transformations can be done as well as additional simple equations can be used. We are sure that over the course of the years this algorithm will be further refined. Our intention to go in this direction and we are going to extend and to apply SEsM for obtaining solutions of many nonlinear differential equations of practical significance for natural and social sciences.
